# Dorsal pulvinar inactivation leads to spatial selection bias without perceptual deficit

**DOI:** 10.1038/s41598-024-62056-5

**Published:** 2024-06-04

**Authors:** Kristin Kaduk, Melanie Wilke, Igor Kagan

**Affiliations:** 1 Decision and Awareness Group, Cognitive Neuroscience Laboratory, German Primate Center, Leibniz Institute for Primate Research, Kellnerweg 4, 37077 Göttingen, Germany; 2 Department of Cognitive Neurology, University of Goettingen, Robert-Koch-Str. 40, 37075 Göttingen, Germany; 3 Cognitive Neurology Group, Cognitive Neuroscience Laboratory, German Primate Center, Leibniz Institute for Primate Research, Kellnerweg 4, 37077 Göttingen, Germany; 4 Leibniz ScienceCampus Primate Cognition, Kellnerweg 4, 37077 Göttingen, Germany; 5 Department of Psychiatry and Psychotherapy, Tübingen Center for Mental Health, University of Tübingen, Tübingen, Germany

**Keywords:** Perceptual decision, Eye movements, Distractors, Spatial choice, Macaque, Decision, Perception

## Abstract

The dorsal pulvinar has been implicated in visuospatial attentional and perceptual confidence processing. Pulvinar lesions in humans and monkeys lead to spatial neglect symptoms, including an overt spatial saccade bias during free choices. However, it remains unclear whether disrupting the dorsal pulvinar during target selection that relies on a perceptual decision leads to a perceptual impairment or a more general spatial orienting and choice deficit. To address this question, we reversibly inactivated the unilateral dorsal pulvinar by injecting GABA-A agonist THIP while two macaque monkeys performed a color discrimination saccade task with varying perceptual difficulty. We used Signal Detection Theory and simulations to dissociate perceptual sensitivity (d-prime) and spatial selection bias (response criterion) effects. We expected a decrease in d-prime if dorsal pulvinar affects perceptual discrimination and a shift in response criterion if dorsal pulvinar is mainly involved in spatial orienting. After the inactivation, we observed response criterion shifts away from contralesional stimuli, especially when two competing stimuli in opposite hemifields were present. Notably, the d-prime and overall accuracy remained largely unaffected. Our results underline the critical contribution of the dorsal pulvinar to spatial orienting and action selection while showing it to be less important for visual perceptual discrimination.

## Introduction

Visual scenes contain multiple spatial locations that serve as potential saccade targets. Selecting a target in a complex scene requires efficiently perceiving and evaluating behaviorally relevant information at different spatial locations. Many studies investigating the neural processes of visuospatial target selection emphasize interactions in frontoparietal cortical networks^1–3^. These direct cortical connections are paralleled by indirect routes through higher-order thalamic nuclei such as the pulvinar, raising the question of how the pulvinar contributes to the selection of behaviorally relevant stimuli to guide visuospatial decision-making^4,5^.

The primate pulvinar consists of several nuclei—anterior, medial, lateral and inferior—with distinct functional properties and connectivity profiles. The dorsal part of the pulvinar (dPul), the focus of the current study, encompasses the anterior and the medial pulvinar and the dorsal part of the lateral pulvinar^6–9^. These nuclei developed together with the association cortices in the course of primate evolution and are reciprocally connected to the parietal and prefrontal cortex, orbitofrontal cortex, insula, cingulate, superior and inferior temporal cortex^10–12^. This extensive connectivity profile identifies the dorsal pulvinar as a unique brain hub well situated to interact with and modulate the circuitry involved in spatial attentional orienting and target selection^13–18^. Studies in human patients with unilateral thalamic lesions encompassing the pulvinar demonstrated deficits related to orienting or responding to perceptually or behaviorally salient stimuli in the contralesional hemifield^19–23^. Similar to neglect/extinction in patients, monkey studies using targeted reversible unilateral pharmacological inactivation of the dorsal pulvinar induced contralesional visuospatial deficits^24–28^. Such deficits manifest behaviorally in several ways. Firstly, the inactivation causes impairment of spatial attentional orienting to cued targets in contralesional hemifield, decreasing performance in detection or color-contingent manual response tasks^24,26^. Secondly, the confidence about contralesional perceptual categorization but not the categorization itself has been reported to decrease after the inactivation, in the absence of competing distractors in the opposite hemifield^25^. Thirdly, a target selection bias away from contralesional hemifield was observed in a free-choice saccade task^27,28^. Such inactivation-induced bias could be alleviated by presenting only a single target or increasing the reward for contralesional targets but less so by perceptual saliency manipulations^27^.

The above causal perturbation findings, and the results of electrophysiological recordings^29–35^, on the one hand, implicate dPul in attentional allocation for perceptual processing, but on the other, are also compatible with a role in more general spatial orienting and selection bias. Different task demands might be one reason for such interpretational ambiguity. In particular, studies that used attentional cueing and perceptual discrimination employed paradigms where manual responses (e.g. button presses) were dissociated from the spatial position of the visual stimuli. At the same time, our previous choice tasks always required a saccade towards a peripheral target and did not involve difficult perceptual discrimination^27,28,31^. Therefore, it remains unclear if perceptual factors contribute to contralesional visuospatial deficits under conditions of spatial competition and target-congruent saccade actions.

In the current study we used a color discrimination saccade selection task to address this question with two essential features. Firstly, we included easy and difficult (i.e. perceptually similar to a target) distractors that should not be selected with a saccade. Secondly, we introduced an option to maintain central fixation as a correct response when only distractor(s) were presented. The task involved three different stimulus types—single stimuli, double “same” stimuli (target-target or distractor-distractor) and double different stimuli. Single stimuli included a peripheral target or a distractor and a central fixation option, resulting in low spatial competition. The double stimuli included left and right peripheral stimuli and a fixation option, adding competition between hemifields. Using Signal Detection Theory^36,37^, we investigated whether unilateral dPul inactivation leads to a perceptual deficit or a spatial selection bias, similarly to recent cortical and superior colliculus studies^38–41^. Suppose the dorsal pulvinar is mainly involved in spatial orienting. In that case, we expect a shift in the criterion manifesting as a selection bias away from the contralesional hemifield, regardless of whether a target or a distractor is presented. But if the dorsal pulvinar is involved in discriminating targets from distractors, we expect a contralesional perceptual sensitivity deficit, manifesting as a decrease in d-prime. Furthermore, if dPul is mainly relevant for regulating the competition between hemifields, we expect a more substantial effect of inactivation in double stimuli conditions.

## Results

**Figure 1:**
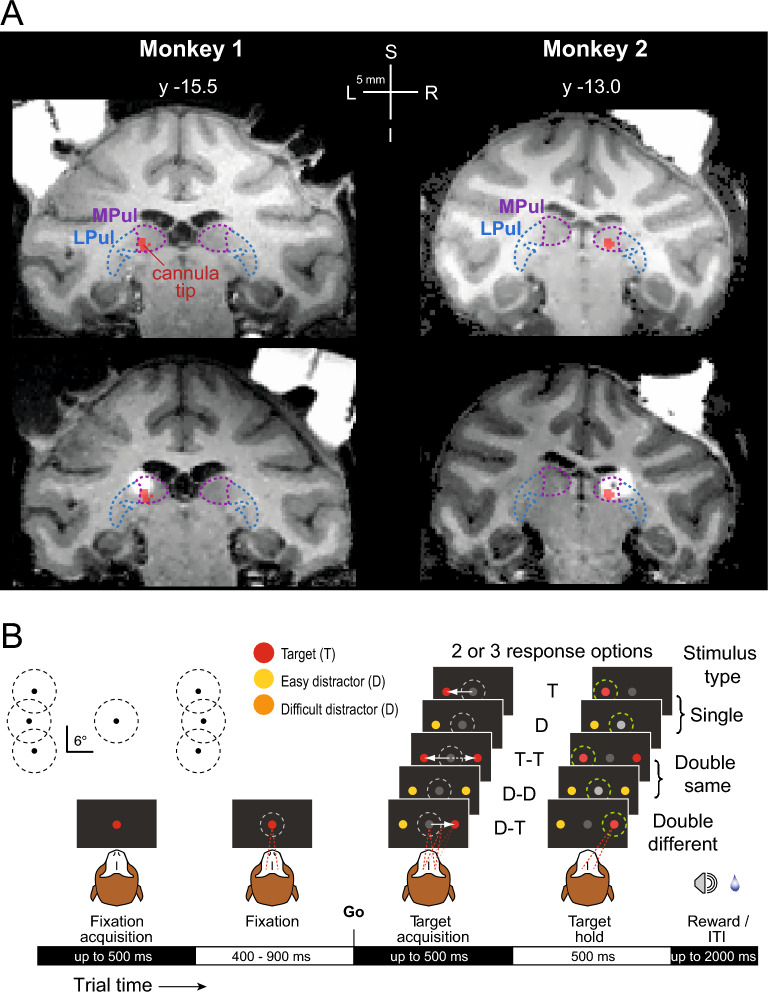
Inactivation sites and task design. (**A**) Top row: MRI coronal sections in the anterior commissure-posterior commissure space (AC-PC, the y coordinate denotes the distance from the AC in mm; note that due to a smaller size of M2’s brain the y coordinate appears less posterior but is in fact located in a similar anterior–posterior location of the pulvinar as in M1). Overlaid borders of medial pulvinar (MPul) and lateral pulvinar (LPul), adapted from the NeuroMaps atlas^42^, are shown with reconstructed locations of injection cannula tips (red). Bottom row: MR images showing the inactivation sites visualized with the injection of MRI contrast agent gadolinium (white), ~ 20 min following the injection (for M1: 2 µl and M2: 3 µl, respectively), 1–2 mm shallower than the final injection locations. (**B**) Color discrimination saccade task where the perceptual difficulty was determined by the color similarity of the target (T, red) vs. distractor (D, easy—yellow, difficult—orange). Target or distractor was presented alone or with a second stimulus (distractor or target) in the opposite hemifield. Monkeys had to saccade to a target after the Go signal or continue fixating when only distractor(s) were presented. Green dashed circles in the target hold period denote correct and rewarded responses. The inset on the top left shows the actual positions of the stimuli (3 positions on the left and 3 positions on the right) and the fixation radii.

We investigated whether local unilateral injections of GABA-A agonist THIP suppressing the dorsal pulvinar neuronal activity (Fig. 1A) cause a contralesional perceptual discrimination deficit or a spatial selection bias. Two monkeys performed a color discrimination task between red targets and distractors (orange stimuli as difficult distractors and yellow stimuli as easy distractors) in three stimulus type conditions (single stimuli, double same stimuli, double different stimuli, Fig. 1B). We analyzed the following dependent variables: saccade latency, accuracy, d-prime (sensitivity), and response criterion (spatial choice bias). The main statistical analysis focused on the comparison between control vs. inactivation sessions, and data were pooled across three left stimulus positions and the three right stimulus positions, for the analysis of contralesional and ipsilesional hemifield effects.

### The effects of dorsal pulvinar inactivation on saccade latency

The saccade latency is a sensitive measure of the effect of dorsal pulvinar inactivation. According to previous work, we expected either faster ipsilesional saccades^28^ or/and slower contralesional saccades^27^. A three-way mixed ANOVA (see details in Suppl. Table S1) was performed to compare the effect of the within-factors “Stimulus Type” (Single/Double Same/Double Different) and “Hemifield” (Contra-/Ipsilateral hemifield) and the between-factor “Perturbation” (Control/Inactivation sessions) on saccade latency. We found a statistically significant Stimulus type × Hemifield × Perturbation interaction for both monkeys (3-way interaction: M1: F(2,22) = 4.64, *p =* 0.021; M2: F(2,24) = 3.74, *p =* 0.039), as well as the Hemifield × Perturbation interaction for M1 (2-way interaction: M1: F(1,11) = 21.81, *p =* 0.001; M2: F(1, 12) = 3.74, *p =* 0.08).

**Figure 2:**
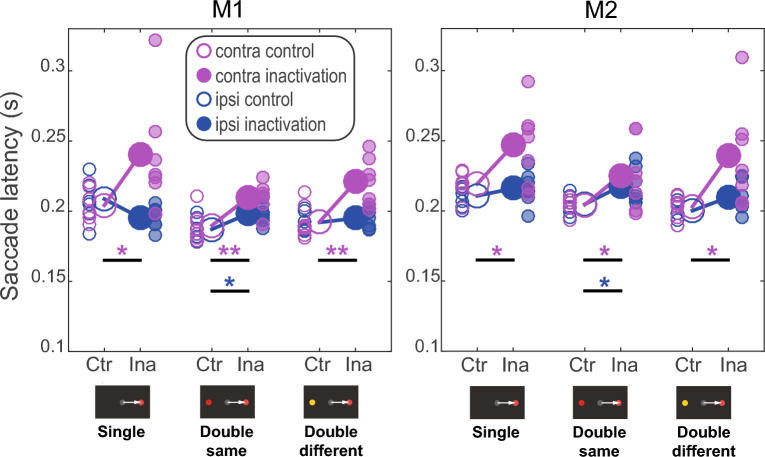
Inactivation effects on saccade latency to the target. The saccade latency is displayed separately for control (empty circles, “Ctr”) and inactivation (filled circles, “Ina”) sessions for each stimulus type and hemifield (icons below the horizontal axis show example stimulus display for one hemifield). Small circles display single sessions; large circles display the mean across sessions. We tested the difference between control and inactivation saccade latency of selecting either contralesional (magenta) or ipsilesional (blue) stimuli (independent t-test; one star *p < 0.05; two stars **p < 0.01).

In agreement with our expectations about the inactivation effects, both monkeys significantly slowed down after dorsal pulvinar inactivation during contralesional target selection for both difficulty levels in all three stimulus types (independent t-test, Table 1, Fig. 2). The consistent inactivation effect across stimulus types and difficulty levels on the contralesional saccade latency indicated a successful pharmacological manipulation. The saccade latency effects for ipsilesional target selection were less pronounced and only reached significance for the double same stimuli (independent t-test, Table 1, Fig. 2). The inactivation only mildly affected the saccade endpoint accuracy, leading to a minor undershooting in particular in the contralesional hemifield (Suppl. Figure S1).

**Table 1:** Results of the independent t-test for the comparison of saccade latency in control versus inactivation sessions.

Stimulus type	Hemifield	Monkey 1 (M1)		Monkey 2 (M2)	
		t-value	p-value	t-value	p-value
Single stimuli	**contra**	**2.31**	**0.039**	**2.47**	**0.029**
	ipsi	2.07	0.061	0.99	0.342
Double same stimuli	**contra**	**3.21**	**0.008**	**2.19**	**0.049**
	**ipsi**	**2.81**	**0.016**	**2.43**	**0.032**
Double different stimuli	**contra**	**3.62**	**0.004**	**2.54**	**0.026**
	ipsi	0.80	0.44	1.91	0.081

The significant effects are in bold font.

### The effects of stimulus type and inactivation on accuracy

**Figure 3:**
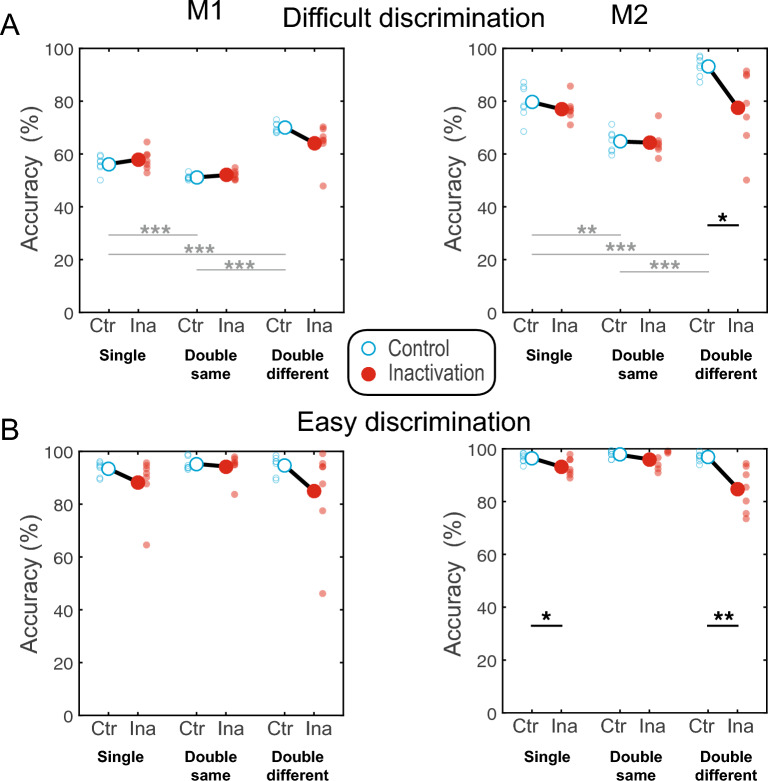
Inactivation effects on accuracy. The accuracy is displayed separately for control (“Ctr”) and inactivation (“Ina”) sessions for each stimulus type and difficulty level. Small circles depict single sessions, and large circles indicate the mean across sessions. Two statistical analyses are presented: the difference between control and inactivation sessions (black connecting lines and stars) and the difference in accuracy between stimulus types (single, double same, double different) for the control sessions (gray connecting lines and stars). (**A**) Difficult discrimination. The inactivation did not affect the accuracy, besides a decrease in the double different condition in M2. Considering only the control sessions, the accuracy significantly varied between stimulus types. (**B**) Easy discrimination. The inactivation affected M2 accuracy in single and double different conditions. The accuracy in the control condition was very high and did not vary between stimulus types. Independent t-test; one star *p < 0.05; two stars **p < 0.01; three stars ***p < 0.001.

The apparent difference between accuracy for easy vs difficult discrimination in the control sessions (Fig. 3) was the intended consequence of our task design (since the accuracy level was manipulated experimentally by adjusting the distractor difficulty, we do not present the corresponding statistical comparisons). However, when we analyzed each difficulty level separately, we also observed accuracy differences between the three stimulus types during difficult discrimination (repeated measures one-way ANOVA, within-factor “Stimulus Type”; for the difficult discrimination: monkey M1: F(2, 20) = 132.26, *p < *0.001; monkey M2: F(2, 20) = 115.96, *p < *0.001; for the easy discrimination: M1: F(2, 20) = 2.23, *p =* 0.15; M2: F(2, 20) = 2.54, *p =* 0.12). In both monkeys, the accuracy was highest for the double different stimuli and lowest in the double same stimuli for the difficult distractor (post-hoc tests; Suppl. Table S2). This difference in accuracy suggests that the three stimulus types elicited different behavioral strategies (for instance, direct comparison between hemifields for double different stimuli vs. only memorized representations of targets and distractors in double same and single stimuli; see later).

We next analyzed the effects of inactivation on accuracy using a three-way mixed ANOVA with within-factors “Stimulus Type” (Single/Double Same/Double Different) and “Difficulty” (Difficult/Easy discrimination) and between-factor “Perturbation” (Control/Inactivation sessions). This analysis (see details in Suppl. Table S3) revealed no main effect of the factor “Perturbation” and no statistically significant interactions with this factor for M1. However, the main effect of the “Perturbation” and the two-way interaction between Stimulus Type × Perturbation was significant for M2 (F(1, 12) = 9.4, *p =* 0.01, F(2,24) = 6.4, *p =* 0.006).

The results in M2 were followed up to investigate the effect of inactivation by applying post-hoc tests. M2 showed a slight but significant decrease in accuracy for single stimuli during easy discrimination (t(1, 12) = − 2.26, *p =* 0.04) and for double different stimuli at both difficulty levels (difficult: t(1, 12) =  − 2.67, *p =* 0.02; easy: t(1, 12) =  − 3.73, *p =* 0.003) but not for double same stimuli (difficult: t(1, 12) = 0.18, *p =* 0.86; easy: t(1, 12) = 1.48, *p =* 0.16) or difficult single stimuli (t(1, 12) = 0.86, *p =* 0.4) (Fig. 3). These effects will be addressed with the Signal Detection Theory analysis below.

### The effects of inactivation on criterion and d-prime

We adopted the Signal Detection Theory approach to differentiate between the spatial selection bias by calculating the response criterion and the deficit in perceptual discrimination between stimuli by calculating d-prime. To assess at first all possible interaction effects on criterion and d-prime, a four-factor mixed ANOVA was used per monkey, including within-factors “Stimulus Type” (Single/Double Same/Double different), “Difficulty” (Difficult/Easy discrimination) and “Hemifield” (Contra-/Ipsilesional) and between-factor “Perturbation” (Control/Inactivation sessions). This ANOVA revealed that there was a significant interaction of all four factors (Perturbation × Hemifield × Difficulty × Stimulus Type) only in M1 for the d-prime (F(2,24) = 3.87, *p =* 0.035) but not for the criterion (F(2,24) = 8.69, *p =* 0.116), and not in M2 (criterion: F(2,24) = 0.33, *p =* 0.72, d-prime: F(2,24) = 0.33, *p =* 0.72). Related to the perturbation, for the criterion M1 showed a three-way Perturbation × Difficulty × Stimulus Type interaction (F(2,24) = 8.69, *p =* 0.001), and two two-way interactions (Perturbation × Difficulty, F(1, 12) = 17.86, *p =* 0.001 and Perturbation × Stimulus Type, F(2,24) = 17.30, *p < *0.001); and for the d-prime, a three-way Perturbation × Hemifield × Stimulus Type interaction (F(2,24) = 3.99, *p =* 0.032). Likewise, M2 showed two two-way interactions (Perturbation × Stimulus Type, F(2,24) = 7.82, *p =* 0.002; Perturbation × Hemifield, F(2,24) = 6.71, *p =* 0.024), and a main effect of Perturbation (F(1, 12) = 12.14, *p =* 0.005) for the criterion; and for the d-prime, a two-way Stimulus Type × Perturbation interaction (F(2,24) = 8.76, *p =* 0.001), and a main effect of Perturbation (F(1, 12) = 10.00, *p =* 0.008) (see the details in the in Suppl. Table S4).

Although the four-factor mixed ANOVA includes all possible interactions, it is difficult to interpret and it cannot directly answer our research question, such as whether dPul inactivation affects the criterion or d-prime, differently for the three stimulus types and the two perceptual difficulty levels. In both monkeys, we observed interactions of the factors “Perturbation” and “Hemifield”, and we had a priori hemifield-specific predictions. To test these predictions, in the sections below we continue the analysis of d-prime and criterion focusing on a two-factor mixed ANOVA (see the details in the Suppl. Table S5 and S6) with the within-factor “Hemifield” (Contra-/Ipsilesional) and the between-factor “Perturbation” (Control/Inactivation sessions), plus the corresponding post-hoc t-tests, separately for each stimulus type and difficulty.

### The effects of inactivation for single stimuli

The single stimuli condition involves a perceptual judgment between making a saccade to a peripheral target or continuing fixating as the correct response to a peripheral distractor (low spatial competition between a central “stay” and peripheral “go” options). To evaluate a deficit in discrimination versus a spatial selection bias, we divided trials into hits, misses, correct rejections, and false alarms separately for stimuli presented in the contralesional hemifield (opposite to the side of inactivation) or ipsilesional hemifield to calculate the hit rate, false alarm rate, d-prime and criterion for each hemifield (Suppl. Figure S2). Since we used two distractors—an easy distractor (yellow) that was perceptually clearly different from the red target and a less apparent (orange) distractor that required a difficult perceptual discrimination, we analyzed these two distractor conditions separately, contrasting them to the target trials. Here and in the next sections, we first describe quantitative predictions using simulated data and then the actual data, separately for difficult and easy discrimination.

Numerical simulations were used to quantify and visualize the relationships between different performance variables under different scenarios of stimulus selection corresponding to the two alternative hypotheses: response bias and perceptual sensitivity deficit, as well as “go/stay” bias (“Methods”). The proportions of hits, misses, correct rejections, and false alarms in a fixed number of trials were defined for the control condition. Next, specific “bias” or “perceptual deficit” on these proportions was introduced to calculate these measures, and resulting criterion and d-prime values in the “inactivated” condition.

**Figure 4:**
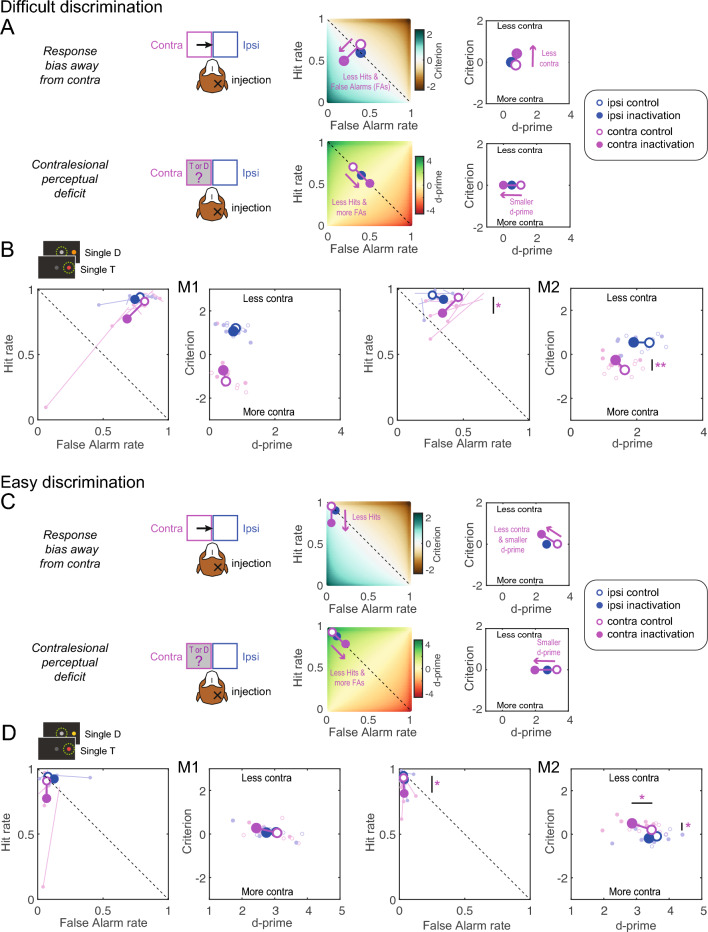
Predictions and results for single stimuli. (**A**) Illustration of the two alternative hypotheses for the difficult discrimination with simulated data showing the expected changes in hit rate, false alarm rate, d-prime and criterion after unilateral dPul inactivation. The color-coded background in the hit rate vs false alarm rate plots shows the corresponding criterion or d-prime. Positive shifts of the criterion are defined as towards “Less contra” (and vice versa). (**B**) Inactivation effects on signal detection variables for the difficult discrimination (orange distractor); the data are displayed separately for each monkey, the left panel shows ipsilesional (blue) and contralesional (magenta) false alarm rate and hit rate, the right panel shows the ipsilesional and contralesional criterion and d-prime. Small circles denote single sessions; large circles –mean across sessions. (**C**) Illustration of the two alternative hypotheses for the easy discrimination. (**D**) Inactivation effects on signal detection variables for the easy discrimination (yellow distractor). Abbreviations: T—target, D—distractor, contra–contralesional, ipsi–ipsilesional.

During difficult discrimination, if dorsal pulvinar inactivation causes a spatial selection bias, we expect a similar decrease in contralesional hit rate and false alarm rate, resulting in a criterion shift towards “less contra” (Fig. 4A). If the inactivation causes a contralesional perceptual discrimination deficit, we expect a decrease in the contralesional hit rate and an increase in the false alarm rate, resulting in decreased contralesional d-prime (Fig. 4A). We did not expect changes to the ipsilesional criterion and d-prime.

In the target trials, the contralesional hit rate decreased after the inactivation, significantly for M2 (independent t-test; M1: t(1, 12) = 1.1, *p =* 0.29; M2: t(1, 12) =  − 2.89, *p =* 0.01). For difficult discrimination (Fig. 4B), the contralesional false alarm rate also decreased, but this effect did not reach significance (M1: t(1, 12) = 1.15, *p =* 0.27; M2: t(1, 12) = 1.6, *p =* 0.14). The two-way mixed-effect ANOVA with factors “Perturbation” and “Hemifield” performed on d-prime and criterion showed no significant interaction of “Perturbation” × “Hemifield” in either monkey, and only a significant main effect of “Perturbation” in M2 on criterion (F(1, 12) = 6.40, *p =* 0.026; see Suppl. Table S5). Accordingly, M2 showed a significant shift of the contralesional criterion towards “less contra” (M2: t(1, 12) = 3.07, *p =* 0.01; the effect was similar but did not reach significance in M1 (t(1, 12) = 1.21, *p =* 0.25). Neither monkey showed a decrease in contralesional d-prime after inactivation (M1: t(1, 12) =  − 0.36, *p =* 0.73; M2: t(1, 12) =  − 1.26, *p =* 0.23). In line with our predictions, in both monkeys neither ipsilesional d-prime nor the ipsilesional criterion exhibited any changes (d-prime M1: t(1, 12) =  − 0.41, *p =* 0.69; M2: t(1, 12) = − 1.98, *p =* 0.07; criterion M1: t(1, 12) =  − 0.99, *p =* 0.34; M2: t(1, 12) = 0.15, *p =* 0.89).

During easy discrimination, if dorsal pulvinar inactivation causes a spatial selection bias, we expect a decrease in the contralesional hit rate but now no change in false alarm rate due to a “floor effect” (already very low false alarm rate in the control sessions). This will result in a shift of criterion towards “less contra”, but importantly, combined with a decrease in the contralesional d-prime (Fig. 4C). For completeness, if the inactivation causes a contralesional perceptual discrimination deficit, one expects a decrease in contralesional hit rate and an increase in false alarm rate (although given the easy discriminability of the yellow distractor, we did not expect such an increase in the actual behavior). This would result in decreased contralesional d-prime but no change in criterion (Fig. 4C).

Indeed, in both monkeys during easy discrimination (Fig. 4D), the false alarm rate was already near zero, so there was no room to exhibit any inactivation-induced decrease (independent t-test; contra M1: t(1, 12) = 0.02, *p =* 0.98; M2: t(1, 12) =  − 0.3, *p =* 0.7; ipsi M1: t(1, 12) = 0.95, *p =* 0.36; M2: t(1, 12) =  − 0.6, *p =* 0.6). In a two-way mixed-effect ANOVA on d-prime and criterion, there was no significant main effect for “Perturbation” or any interaction between “Perturbation” and “Hemifield” in both monkeys (in M2, the interaction for the criterion showed a trend, F(1, 12) = 4.59, *p =* 0.053, see Suppl. Table S6). Accordingly, M2 showed a significant shift for the contralesional criterion towards “less contra” (M1: t(1, 12) = 0.85, *p =* 0.41; M2: t(1, 12) = 2.63, *p =* 0.02), but also a decrease in contralesional d-prime (M1: t(1, 12) =  − 1.49, *p =* 0.16; M2: t(1, 12) = 2.27, *p =* 0.04). This decrease in d-prime was due to the “floor effect” of already very low false alarm rate, as predicted in the simulation.

To sum up, under conditions of low spatial competition, after inactivation both monkeys showed a shift in response criterion manifesting as reluctance to select stimuli in the contralesional hemifield for both difficulty levels, significant in monkey M2.

### The effects of inactivation for double same stimuli

Previous studies suggested that dorsal pulvinar becomes most relevant in the case of spatial competition between hemifields^24,27,31^. Here, two equally rewarded targets or two distractors were presented in the periphery during the double same stimuli condition, eliciting a high competition between hemifields for visual representation and response selection. The monkeys chose between continuing fixating or making a saccade to one of the two peripheral stimuli (Suppl. Figure S3).

**Figure 5:**
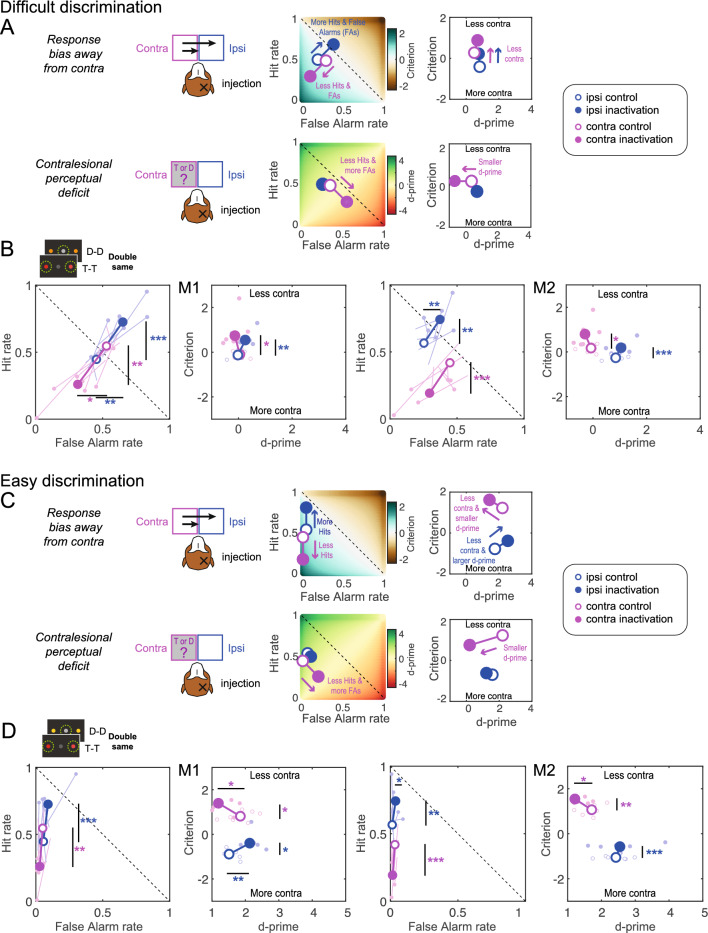
Predictions and results for double same stimuli. Same format and notations as in Fig. 4. (**A**) Illustration of the two alternative hypotheses for the difficult discrimination in case of the double same stimuli. (**B**) Inactivation effects on signal detection variables for the difficult discrimination, with data separately shown for each monkey, with single sessions and overall means displayed. (**C**) Illustration of the two alternative hypotheses for the easy discrimination in case of the double same stimuli. (**D**) Inactivation effects on signal detection variables for the easy discrimination. Abbreviation: T—target, D—distractor, contra–contralesional, ipsi–ipsilesional.

As for the single stimuli, if dorsal pulvinar inactivation causes a spatial selection bias during inter-hemifield competition, we expect a similar decrease in contralesional hit and false alarm rates. Necessarily, such a decrease has to result in either (i) a corresponding increase in ipsilesional hit rate and false alarm rate, or (ii) an increase of central fixation selection. Both monkeys tended to select peripheral stimuli over the central fixation, even after inactivation, as shown with a non-hemifield-selective criterion analysis (“stay” vs. “go”, Suppl. Table S7). Therefore, we expected a shift for both contralesional and ipsilesional criteria towards “less contra” (Fig. 5A). If dorsal pulvinar inactivation causes a contralesional perceptual discrimination deficit, similarly to the single stimuli condition we expect a decrease in contralesional hit rate and an increase in contralesional false alarm rate, resulting in a reduction of contralesional d-prime (Fig. 5A). We did not have strong predictions for the ipsilesional discrimination: it might remain unaffected, as for single stimuli (and instead, only fixation selection might change to counterbalance the contralesional changes, the possibility we illustrate here), or it might improve as a consequence of ipsilesional hit rate increase and ipsilesional false alarm rate decrease.

The contralesional hit rate decreased significantly for both monkeys (independent t-test; M1: t(1, 12) = − 4.24, *p < *0.001; M2: t(1, 12) = − 4.78, *p < *0.001), and ipsilesional hit rate increased significantly (M1: t(1, 12) = 4.36, *p < *0.001; M2: t(1, 12) = 3.28, *p =* 0.01). For difficult discrimination displayed in Fig. 5B, the ipsilesional false alarm rate significantly increased for both monkeys (M1: t(1, 12) = 3.25, *p =* 0.01; M2: t(1, 12) = 3.23, *p =* 0.01), and contralesional false alarm rate significantly decreased for M1 (M1: t(1, 12) =  − 2.79, *p =* 0.02; M2: t(1, 12) =  − 2.04, *p =* 0.065). The two-way mixed-effect ANOVA performed on d-prime and criterion showed no significant “Perturbation” × “Hemifield” interaction (see Suppl. Table S5). For the criterion, a significant main effect for “Perturbation” was observed for both monkeys (M1: F(1, 12) = 9.65, *p =* 0.009; M2: F(1, 12) = 10.43, *p =* 0.007). Both monkeys showed a significant shift of the contralesional and ipsilesional criterion towards “less contra” (M1 contra: t(1, 12) = 2.68, *p =* 0.02, ipsi: t(1, 12) = 3.74, *p =* 0.003; M2 contra: t(1, 12) = 2.87, *p =* 0.014, ipsi: t(1, 12) = 3.8, *p =* 0.003). Neither monkey showed a significant change in contralesional d-prime (*p* > 0.05).

During easy discrimination, as for the single stimuli, for the spatial selection bias hypothesis we expect a decrease in contralesional hit rate and no observable decrease in false alarm rate (again due to the “floor effect” on already very low false alarm rate), and a corresponding increase in ipsilesional hit rate, but no increase in false alarm rate, due to easy discriminability of the distractor. These changes should result in a shift for both contralesional and ipsilesional criteria towards “less contra” combined with a change in d-prime values (Fig. 5C). For the contralesional perceptual discrimination deficit hypothesis, we expect a decrease in the contralesional hit rate and an increase in the false alarm rate resulting in a decrease in contralesional d-prime (Fig. 5C).

For the criterion, we found a significant main effect for “Perturbation” in both monkeys (M1: t(1, 12) = 8.30, *p =* 0.014; M2: t(1, 12) = 31.20, *p < *0.001). For the d-prime, we found significant interaction of “Perturbation” × “Hemifield” in M1 (F(1, 12) = 11.99, *p =* 0.005; Suppl. Table S6). To further evaluate the selection behavior for easy discrimination, we examined the results of the follow-up tests and compared them with the hypothesis-driven simulations. For easy discrimination (Fig. 5D), the false alarm rate was already near zero, so there was no room to exhibit any inactivation-induced decrease: the contralesional false alarm rate did not show an effect (independent t-test; M1: t(1, 12) = − 0.94, *p =* 0.2; M2: t(1, 12) = 1.82, *p =* 0.09). The ipsilesional false alarm rate slightly increased, significant for one monkey (M1: t(1, 12) = 1.37, *p =* 0.37; M2: t(1, 12) = 2.32, *p =* 0.04). Consequently, both monkeys showed a significant shift for the contralesional and ipsilesional criterion towards “less contra” (M1 contra: t(1, 12) = 2.83, *p =* 0.015, ipsi: t(1, 12) = 2.66, *p =* 0.021; M2 contra: t(1, 12) = 3.6, *p =* 0.004, ipsi: t(1, 12) = 7.66, *p < *0.001). Both monkeys also showed a significant decrease in contralesional d-prime (M1: t(1, 12) = − 2.32, *p =* 0.039; M2: t(1, 12) = − 2.30, *p =* 0.04), due to the already very low false alarm rate, and M1 showed an increase in ipsilesional d-prime (M1: (1, 12) = 3.31, *p =* 0.006, M2: (1, 12) = − 0.38, *p =* 0.71), due to increase in ipsilesional hit rate but without corresponding increase in ipsilesional false alarm rate. Overall, the data for both difficulty levels are consistent with the spatial selection bias hypothesis.

### The effects of inactivation for double different stimuli

Similar to the double same stimuli, the double different stimuli condition also comprises a high spatial competition between hemifields. Furthermore, it might be influenced by the possibility of directly comparing the simultaneously presented target and distractor in the opposite hemifields. Notably, the central fixation is always an incorrect response option in this condition (Suppl. Figure S4).

**Figure 6:**
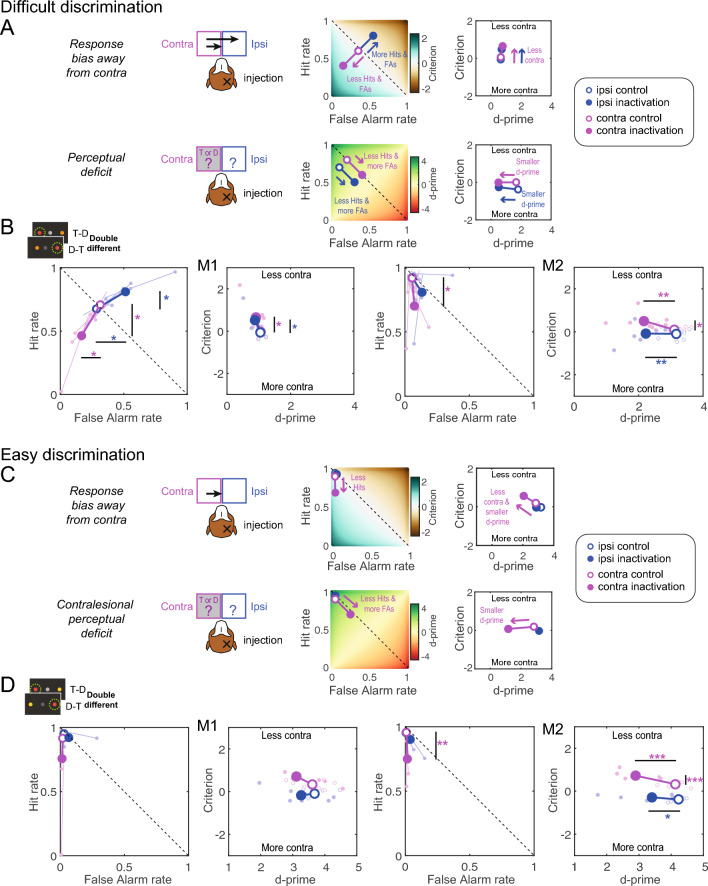
Predictions and results for double different stimuli. Same format and notations as in Fig. 4. (**A**) Illustration of the two alternative hypotheses for the difficult discrimination in case of the double different stimuli. (**B**) Inactivation effects on signal detection variables for the difficult discrimination, with data separately shown for each monkey, with single sessions and overall means displayed. (**C**) Illustration of the two alternative hypotheses for the easy discrimination in case of the double different stimuli. (**D**) Inactivation effects on signal detection variables for the easy discrimination. Abbreviation: T—target, D—distractor, contra–contralesional, ipsi–ipsilesional.

During difficult discrimination, for the spatial selection bias hypothesis we expect the same effects as for the double same stimuli (Fig. 6A). But for the perceptual discrimination deficit hypothesis, assuming a persistent “go” bias, in contrast to double same stimuli here we expect the decrease in contralesional d-prime to be necessarily linked to the *decrease* in ipsilesional d-prime. This is because selecting less targets on the contralesional side would lead to selecting *more* distractors on the ipsilesional side, and selecting more contralesional distractors—to *less* ipsilesional targets (Fig. 6A).

During difficult discrimination, the contralesional hit rate decreased significantly in both monkeys (independent t-test; M1: t(1, 12) =  − 2.8, *p =* 0.02; M2: t(1, 12) = − 2.98, *p =* 0.01) and ipsilesional hit rate increased for M1 (M1: t(1, 12) = 2.92, *p =* 0.01; M2: t(1, 12) = 1.49, *p =* 0.16; Fig. 6B). The contralesional and ipsilesional false alarm rate significantly decreased only for M1 (contra: M1: t(1, 12) =  − 2.9, *p =* 0.01; M2: t(1, 12) =  − 0.65, *p =* 0.53; ipsi: M1: t(1, 12) =  − 2.9, *p =* 0.01; M2: t(1, 12) =  − 1.8, *p =* 0.09). Consequently, we observed for the criterion a significant main effect of “Perturbation” in M1 (F(1, 12) = 6.47, *p =* 0.026) and an interaction of “Perturbation” × “Hemifield” in M2 (M2: F(1, 12) = 6.88, *p =* 0.022; Suppl. S5). In line with the response bias hypothesis, M1 showed a significant shift for the contralesional and ipsilesional criterion towards “less contra” (contra: t(1, 12) = 2.4, *p =* 0.03, ipsi: t(1, 12) = 2.72, *p =* 0.02) and no effect for contralesional or ipsilesional d-prime (contra: t(1, 12) =  − 1.42, *p =* 0.18, ipsi: t(1, 12) =  − 2.03, *p =* 0.06). Likewise, M2 showed a significant shift for the contralesional criterion towards “less contra” (M2: t(1, 12) = 2.42, *p =* 0.03). But, in accordance with a significant main effect of “Perturbation” for the d-prime in M2 (F(1, 12) = 10.73, *p =* 0.007), M2 also exhibited a significant decrease in contralesional and ipsilesional d-prime (contra: t(1, 12) =  − 3.14, *p =* 0.01; ipsi: t(1, 12) =  − 3.32, *p =* 0.006).

During easy discrimination in the presence of a yellow distractor, for the spatial selection bias hypothesis, we expect a decrease in a contralesional hit without the decrease in false alarm rate due to the “floor effect”. We also expect no increase in the ipsilesional hit rate because it is already very high and no increase in the ipsilesional false alarm rate because of the easy discriminability of the yellow distractor (Fig. 6C). For contralesional perceptual discrimination deficit, similarly to the double same stimuli, we expect a decrease in contralesional d-prime but no effect on ipsilesional d-prime (Fig. 6C).

For easy discrimination (Fig. 6D), the false alarm rate was already near zero, so there was no room to exhibit any inactivation-induced contralesional decrease (independent t-test; M1: t(1, 12) = 0.8, *p =* 0.47; M2: t(1, 12) =  − 1.87, *p =* 0.09). The ipsilesional false alarm rate did not increase (M1: t(1, 12) = 0.98, *p =* 0.35; M2: t(1, 12) = 1.66, *p =* 0.12). The contralesional hit rate decreased for M2 (M1: t(1, 12) =  − 1.2, *p =* 0.25; M2: t(1, 12) =  − 4.22, *p < *0.001). In the ANOVA, for the criterion we found a significant interaction of “Perturbation” × “Hemifield”, and the main effect of “Perturbation” in M2 but not M1 (F(1, 12) = 9.52, *p =* 0.009; F(1, 12) = 22.96, *p < *0.001; Suppl. Table S6). Accordingly, only M2 showed a significant shift for the contralesional criterion towards “less contra” (M1: t(1, 12) = 0.82, *p =* 0.25; M2: t(1, 12) = 4.75, *p < *0.001). Furthermore, in M2 there was a significant main effect of “Perturbation” for the d-prime (F(1, 12) = 13.49, *p =* 0.003) and a corresponding decrease in contralesional and ipsilesional d-prime (contra M1: t(1, 12) =  − 0.82, *p =* 0.43; M2: t(1, 12) =  − 5.16, *p < *0.001; ipsi: M1: t(1, 12) =  − 2.03, *p =* 0.07; M2: t(1, 12) =  − 2.19, *p =* 0.049). This decrease in d-prime was due to the “floor effect” of already very low false alarm rate.

To sum up these results for double different stimuli, the inactivation effects in M1 during difficult discrimination fully matched the predictions of the spatial selection bias hypothesis (shift of both contralesional and ipsilesional criteria towards “less contra”). In contrast, in M2 only the contralesional criterion, for both difficulties, shifted towards “less contra”. In M2, we also observed a decrease in contralesional and ipsilesional d-prime for both difficulty levels. In control sessions, M2 had a very high hit rate and a very low false alarm rate during both easy and difficult discrimination. Therefore, the contralesional d-prime decline can be accounted for by the impossibility of going below the already very low false alarm rate (for both difficulty levels) coupled with a decrease from the initially very high hit rate, similarly to single and double same stimuli conditions for easy discrimination. This suggests that the response bias is the underlying cause of these d-prime changes. But the ipsilesional d-prime decrease, which only manifested in the double different stimuli condition, might be related to the compromised ability of this monkey to utilize the information from the contralesional hemifield for the direct comparison with the ipsilesional stimulus—the strategy that M2 might have successfully relied upon in the control sessions (*cf.* Figure 3 and further arguments in the Discussion).

**Figure 7:**
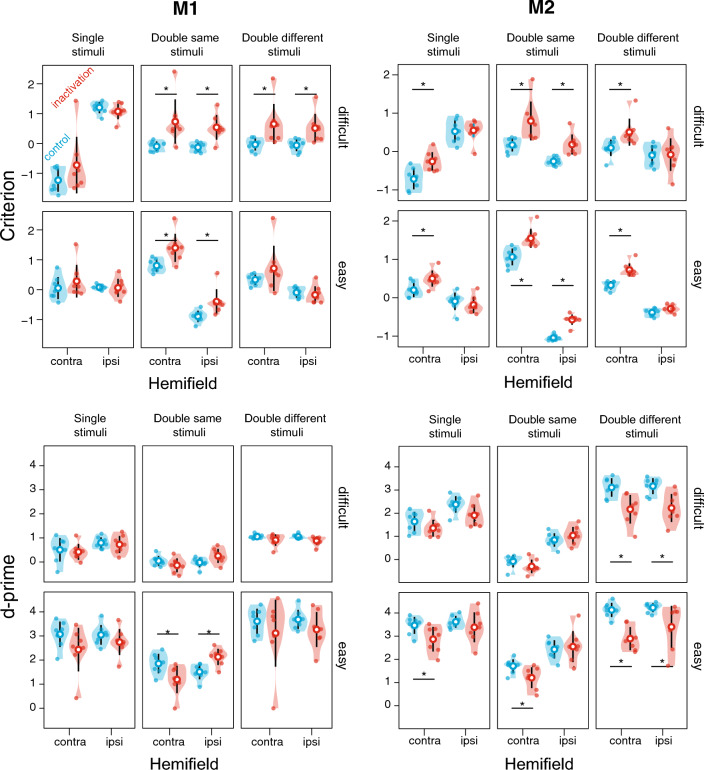
Summary of inactivation effects on criterion and d-prime, for all stimulus conditions. The violin plots display the distribution of the session values for the two difficulty levels (difficult and easy), stimulus types (single/double same/double different stimuli), and hemifield (contra- and ipsilesional) for control (blue) and inactivation (red) sessions. The means are displayed as empty circles and 95% confidence intervals are shown as black vertical lines. Each small dot represents a session. The stars display the significance of the independent t-test comparing control and inactivation sessions at the *p*-value < 0.05. See Table 2 for the statistics.

All stimulus conditions are summarized together in Fig. 7 and Table 2. The consistent effects point to the criterion change due to the spatial bias away from the contralesional hemifield (“less contra”). Overall, the bias appears to be more prominent in M2, and it underlies frequent changes in the d-prime, as mentioned above and addressed further in the Discussion.

**Table 2:**
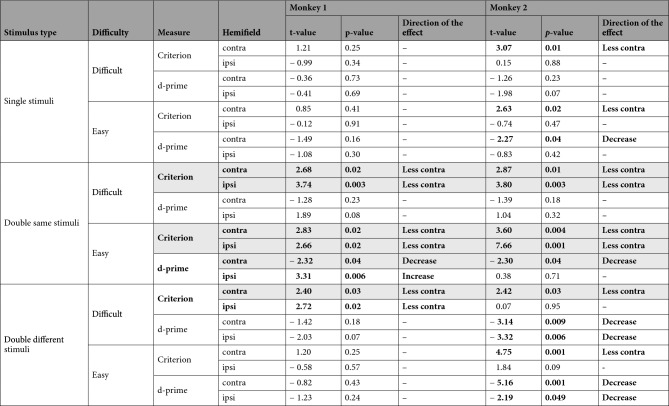
Summary of inactivation effects for criterion and d-prime using independent t-tests.

Significant effects are in bold font, and consistent effects across the two monkeys are highlighted with gray background. Figure 7 plots the summary of the corresponding data, and Suppl. Table S8 shows the results of nonparametric tests.

## Discussion

This study utilized task demands such as fast perceptual color discrimination between target and distractor, spatially competing stimuli, and stimulus-congruent saccade responses to investigate whether the impairments in contralesional perceptual discrimination, as opposed to more general response bias, might contribute to visuospatial deficits after dorsal pulvinar inactivation. Following the inactivation, we primarily observed slowing of contralesional saccades and criterion shifts away from contralesional stimuli, especially when two peripheral stimuli elicited high spatial competition between hemifields (Table 2). These effects were present at both perceptual difficulty levels. Notably, the d-prime and the overall accuracy remained largely unaffected. We conclude that the contralesional visuospatial deficits observed after inactivating the dorsal pulvinar are not caused by a contralesional perceptual deficiency but by a spatial selection bias, even during a demanding perceptual decision task.

We adopted the Signal Detection Theory approach to differentiate between spatial selection bias and discrimination sensitivity by calculating the response criterion and d-prime, as has been done in studies of visual and prefrontal cortices^40,43^ and the superior colliculus^38,39,44^. After pulvinar inactivation, a shift in criterion manifested as reluctance to select stimuli in the contralesional hemifield, regardless of whether it contained a target or a distractor. This spatial selection bias, and delays in making a saccade to contralesional stimuli, were observed for all three stimulus conditions (single, double same, and double different). In our experiments, as in many prior studies, the saccadic “report” coincided with the target stimulus location. Therefore, the observed change in the decision criterion could be attributed to both a (pre)motor “response bias”, as well to a more stimulus position-specific “choice bias” that would also be revealed by a dissociated saccade or even a manual report^38,41,45^. Note, however, that we purposely designed the task to probe pulvinar under ecologically-valid conditions where sensory information aligns with the spatial goals.

Similar to our previous work^27,28^, here we found a pronounced selection bias “away from contra” for the double same stimulus condition most similar to the free-choice between two identical targets. But also for single stimuli, the shift of the criterion “away from contra” was significant in one monkey and similar (but not significant) in the other monkey. In contrast to the current results, prior studies^27,28^ did not find a decrease in the correct selection of contralesional single instructed targets. In these studies, the instructed single target condition differed from the current single stimulus condition because monkeys invariably had to saccade to the target. Here, we included a second response option, staying at the central fixation point, as the correct response for the distractor trials. The presence of two options and the perceptual discrimination task context created a low level of spatial competition between the fixation and the peripheral option in the single stimuli condition—sufficient to engender an effect from pulvinar inactivation. Hence, the dorsal pulvinar inactivation influenced the competition not only between the hemifields but also the competition between the foveal and the peripheral contralesional options.

Support for the importance of spatial competition also comes from a brief report by Desimone and colleagues, who inactivated the lateral pulvinar in one macaque during a color rule task that required a specific response with a manual lever to a briefly flashed colored target and not to a distractor^24^. The target was defined as the stimulus appearing at the same location as the briefly flashed cue. The error rate increased when the cue and the target were in the contralesional hemifield while the conflicting distractor was in the ipsilesional hemifield. However, when both the target and the distractor were located within the same hemifield—thereby obviating the competition between hemifields—pulvinar inactivation did not yield any significant impact on performance.

Notably, in the present study the inactivation-induced selection bias occurred even when only one response option was correct and rewarded. Placing a target in the contralesional hemifield when it was the only rewarded option (i.e. single target or target-distractor conditions) did not alleviate the spatial selection bias “away from contra”. Both monkeys selected the contralesional target less and instead chose the ipsilesional distractor or the fixation option, receiving no reward in these trials. Again, this contrasts with the observed alleviation of the selection bias in the value-based saccade choice task where a color cue explicitly and unambiguously signaled a large reward^27^. Another difference to the present study was that in Wilke and colleagues^27^ there was a memory delay that monkeys could have utilized to make a deliberate value-based choice. The present results emphasize the contribution of dPul to fast decisions between conflicting options under uncertainty, in agreement with the previous inactivation study in the lateral pulvinar^24^, and in agreement with a microstimulation study from our group which showed a strong microstimulation-induced choice bias in the immediate visually-guided saccade task but not in the delayed memory-guided task^31^.

A hypothetical inactivation-induced contralesional perceptual discrimination deficit should engender a confusion between targets and distractors in the contralesional hemifield. This would lead to an increased false alarm rate alongside a decreased hit rate, resulting in a decreased contralesional d-prime. We did not observe a pattern that would be consistent with a contralesional perceptual discrimination deficit in any of the three stimulus type conditions. Likewise, previous pulvinar lesion studies in non-human primates showed unimpaired visual discrimination learning^46,47^ and unimpaired contralesional visual motion discrimination performance without spatially-competing distractors^25^. These studies however used manual responses that were spatially dissociated from the visual stimuli. Our study extends these findings to situations in which saccadic responses are spatially-contingent on the stimuli, and there is a competition between spatial locations. But even under these conditions, where the contribution of the pulvinar might be more crucial, the perceptual sensitivity was largely unaffected after pulvinar inactivation.

One possible strategy to accurately discriminate targets from distractors in our task is to compare the presently visible stimuli with learned and memorized representations of the target and the distractors. This strategy could be used for all three stimulus types. For the double different stimuli condition, however, an additional strategy could be employed. The visual appearance of the two stimuli, one target, and another distractor, could be directly compared across hemifields without relying upon, or in addition to, memorized representations. Indeed, both monkeys had significantly higher accuracy in the target-distractor condition than in the single or double same stimuli conditions for the difficult discrimination. At the same time, the accuracy is shaped by both the hit rate as well as the false alarm rate, and it is plausible to expect that the additional direct comparison strategy will affect both measures. In monkey M2, the hit rates during difficult discrimination were already very high (> 90%) both in the least accurate (single stimuli) and most accurate (double different stimuli) conditions. We speculate that only the false alarm rate had room to decrease in the latter condition due to the additional strategy. Furthermore, in the control sessions the accuracy in both easy and difficult target-distractor trials was similarly high in monkey M2. Hence, we contend that the strategy based on the direct across-hemifield comparison improved discrimination performance for the target-distractor condition in M2. But in monkey M1, compared to the single stimuli, the hit rate decreased—while the false alarm rate decreased even more—in the double different stimuli condition, resulting in a net gain in the accuracy. These effects are less compatible with the additional comparison strategy, and instead indicate a detrimental impact of a stronger “go” bias in the single stimuli condition.

After inactivation, M2’s accuracy decreased substantially for the double different stimuli (target-distractor) condition, driven mainly by a drop in contralesional hit rates (but without the corresponding increase in false alarm rate). Furthermore, there was a significant drop in both, contralesional and ipsilesional d-prime. We argue that this is not an indication of a specific contralesional perceptual discrimination deficit but a consequence of the direct across-hemifield comparison strategy, and its partial failure due to the spatial bias away from the contralesional hemifield. In this interpretation, the drop in the hit rate but not the increase in the false alarm rate is expected. M2 went less often to the contralesional hemifield (due to the criterion shift), but could still utilize the information from the ipsilesional hemifield to correctly reject difficult contralesional distractors, as demonstrated by a very low contralesional false alarm rate even after the inactivation. The resulting contralesional d-prime decrease during difficult discrimination is thus similar to the case of easy discrimination. At the same time, the ipsilesional hit rate decreased while the ipsilesional false alarm rate increased non-significantly. We suggest that the ipsilesional d-prime decreased because the inactivation-induced spatial bias disrupted the access to information from contralesional hemifield for comparing it to the stimulus in the ipsilesional hemifield.

Pulvinar lesions in humans^19,22,48^ and monkeys^24,26,33,49^ lead to deficits in spatial attention tasks. Prior work also showed that subjects may shift either their criterion or sensitivity at the attended location relative to the unattended location^40,43,50^. These studies raise the possibility of relating our findings to visual spatial attention. Experiments investigating spatial attention typically use a valid or invalid cue indicating where to attend to an upcoming target without making an eye movement^26,32,40,41,51,52^. Our task was not designed to investigate covert spatial attentional processes or shifts of attention, as it lacked the attentional cue. Nevertheless, our main finding of the spatial selection bias after dorsal pulvinar inactivation generally fits well with the previous work emphasizing the role of the pulvinar in selective spatial attention^4,53^.

Indeed, spatial choice bias is often considered a component, or a manifestation of attentional processing, because it captures the competition between spatial locations^54,55^. Desimone and Duncan proposed interpreting the findings after the unilateral pulvinar inactivation as formulated in the biased competition theory^56^. In this framework, unilateral inactivation puts the contralesional hemifield at a disadvantage for selection by biasing the ongoing competition for the neuronal representation of multiple stimuli, hence leading to the observed bias “away from contra”. When stimuli compete for attention, attending to one option biases the competition by enhancing the activity in the neurons representing this response option within their receptive field. We speculate that the activity in the pulvinar—and/or in the connected cortical regions—in response to a salient yellow distractor will be suppressed compared to a target or a difficult distractor (target > difficult distractor > easy distractor)—as has been found in V4 during visual search in the presence of a salient pop-out color distractor^57^. After pulvinar inactivation, we expect a decrease in the saliency (and in the underlying activity) of contralesional stimuli—especially when they are not selected—and the corresponding increase in the salience of ipsilesional stimuli due to a push–pull between competing representations^58^. At the same time, we expect that the graded response amplitude for targets and distractors will persist.

Given tight links between attention, perceptual decision and confidence^59,60^, the inactivation-induced changes in contralesional salience or criterion might also lead to an altered confidence in choosing the correct response^61^. Our task did not involve confidence judgments. It is plausible however that decreased contralesional confidence in choosing the correct response, operationalized as the increased frequency of opt-out choices^25^, might result in a spatial selection bias ‘away from contra’ that we have observed. Conversely, the diminished confidence about contralesional stimuli may be a consequence of the observed criterion change. Further studies need to investigate the interplay between response and choice bias, sensitivity, bottom-up or/and top-down saliency of a stimulus, and confidence, by utilizing tasks designed to dissociate between these contributing factors^38,40,41,50,62^.

While in the present study we focused on the dorsal pulvinar, the ventral pulvinar (encompassing the ventral part of the lateral pulvinar, PLvl, and the inferior pulvinar) is also associated with visual attention and salience^16,49,63^. In particular, the inferior pulvinar can be considered a more perceptually-relevant visual nucleus due to its extensive connectivity to the primary visual cortex and the ventral visual stream^10,11,15,64^. Given such connectivity, visual response properties^6,49,65–67^, visual target-related microstimulation effects^31^, and strong perceptual modulation^68^, it would be interesting to investigate if the ventral pulvinar inactivation engenders a more substantial perceptual deficit than the dorsal pulvinar.

In regard to both, the dorsal and the ventral pulvinar, the human neuroimaging and patient literature sometimes describes the function of pulvinar as distractor filtering^23,69^. For instance, LaBerge and Buchsbaum^70^ investigated the contribution of the pulvinar to visual distractor processing using positron emission tomography in healthy subjects. Increased pulvinar activation was found when the target in the contralateral visual field was surrounded by distractors relative to no distractors. The authors concluded that identifying an object in a cluttered visual scene might involve the pulvinar through a filtering mechanism. Similarly, several fMRI studies showed increased activation during more demanding distractor conditions—but these effects could have been driven by task difficulty and response competition rather than specific contralateral filtering processing^71,72^. Likewise, very few patient studies support the notion that the pulvinar participates in filtering out distracting visuospatial inputs in the contralateral hemifield. A study on oculomotor capture found that patients with unilateral pulvinar lesions made slightly more errors when the target was ipsilesional and the distractor was contralesional, compared to the reverse configuration—although the effect was very small (3% error rate difference)^73^. Another study in patients with ventral pulvinar lesions showed impaired contralateral perceptual discrimination in the presence of flanking distractors^48^.

One interpretation of the distractor filtering hypothesis is that unilateral pulvinar inactivation should disrupt monitoring and filtering out the distractors in the *contralesional* hemifield. If so, we should observe an increased selection of contralesional distractors. We however found no inactivation-induced increase in false alarm rate when an easy or a difficult distractor was presented in the contralesional hemifield. An additional counterargument against the contralateral distractor filtering hypothesis can be derived from cued spatial attention tasks. The inactivation of the lateral pulvinar decreased the error rate due to contralesional distractors—i.e. decreased contralesional false alarm rate^24^. Similarly, dorsal lateral pulvinar inactivation caused faster reaction times, compared to control, when the distracting invalid cue was contralesional and the target ipsilesional^26^. Collectively, these observations, in conjunction with our findings, challenge the role of the pulvinar in filtering out contralesional distractors, particularly under conditions where stimuli are isolated and not subject to crowding. Instead, the pulvinar might be crucial for the contralateral spatial orienting and selective attention, as was also suggested in a number of patient studies that typically combined dorsal and ventral pulvinar lesions due to stroke etiology^21,74–76^.

Beyond the pulvinar, an ipsilesional selection bias and/or increased contralesional saccade latencies often result from perturbations of frontoparietal cortex and the superior colliculus. For instance, one or both such effects occur after inactivating the lateral intraparietal area (LIP) during a visual search^77,78^ and memory saccade tasks^79–82^, after inactivating frontal eye fields (FEF) in a visual search task^83^, and after parietal or prefrontal cortex inactivation during stimulus-onset asynchrony saccade task^84,85^. These general similarities are in line with dorsal pulvinar’s reciprocal anatomical^8,86–88^ and functional connections^15,32,65^ with the frontoparietal cortex. In addition to the frontoparietal connectivity, recent work also emphasized the role of the superior temporal regions—which are also strongly interconnected with the pulvinar—in visuospatial processing^89^. Of course, the specifics of impairments differ between the regions and the paradigms. For instance, the reaction time deficits in visual search target detection depend on the perceptual task difficulty after LIP but not after FEF inactivation^83^. In the context of the Signal Detection Theory, while several studies pointed to decision criterion changes after the superior colliculus perturbation^38,44,45^, the effect on perceptual sensitivity has also been demonstrated^39^. A major challenge for future research is to compare specific contributions of the pulvinar with other subcortical structures that mediate selective attention and target selection via thalamo-cortical pathways, such as the superior colliculus and basal ganglia^38,89,90^, for example by using pathway-selective manipulations^91,92^.

## Methods

### Experimental procedures

All experimental procedures complied with the ARRIVE guidelines (https://arriveguidelines.org) and were conducted in accordance with the European Directive 2010/63/EU, the corresponding German law governing animal welfare, and German Primate Center institutional guidelines. The procedures were approved by the responsible government agency (Niedersaechsisches Landesamt fuer Verbraucherschutz und Lebensmittelsicherheit (LAVES), Oldenburg, Germany).

Two adult male rhesus monkeys (*Macaca mulatta*), weighing 10.5 kg and 9 kg, served as subjects (monkey 1, “Cor”, M1; monkey 2, “Cur”, M2). For both monkeys, the surgical procedures for implanting an MRI-compatible headpost and chambers, and small within-chamber craniotomies, were the same as described in Dominguez-Vargas et al.^31^. The inactivation locations in the dorsal pulvinar were estimated based on anatomical MRI as described in more detail in the previous work^15,31^. Based on the MRI images, we planned where to target the dorsal pulvinar (the grid hole and depth) using the software Planner^93^ and BrainVoyager (Version 2.4.2, 64-bit; Brain Innovation). To confirm the inactivation locations, we performed injections of MRI contrast agent gadolinium, diluted 1:200 with saline (Fig. 1A). These control injections were performed 1–2 mm shallower than the final injection depths (Fig. 1A, Suppl. Figure S5).

The neural activity was reversibly suppressed using the GABA-A agonist 4,5,6,7-tetrahydro isoxazole [5,4-c]-pyridine-3-ol (THIP hydrochloride; Tocris). The THIP was dissolved in phosphate-buffered saline (PBS). The solution (pH 7.0–7.5) was sterile filtered with a hydrophobic PTFE membrane filter (pore size: 0.2 µm, Sartorius) before injection via a sterile 50 or 60 mm length 31 gauge sharp-tip steel cannula (Plastics One). The solution was delivered at a rate of 0.25 µl/min using a 0.1 ml glass-tight Hamilton syringe driven by a digital infusion pump (Harvard Apparatus). The injected volume was determined for each monkey separately in pilot sessions, so that after the injection the monkey could perform the task without nystagmus but showed an ipsilesional spatial bias for target-target (“free choice”) stimuli as in prior reports, indicating a successful inactivation procedure^27,28^. In Monkey 2, a strong jerk nystagmus—with a fast phase towards the ipsilesional hemifield—began approximately 40 min after the injection of 4–2.5 µl. This made it difficult to fixate and complete a sufficient number of trials. In Monkey 1, a weaker form of nystagmus occasionally transpired later in the session, after enough trials had already been collected. Hence, the final injection volume per session was 4.5–5 µl of 10 mg/ml of THIP for monkey 1 (dPul in the left hemisphere) and 1.5–2 µl for monkey 2 (dPul in the right hemisphere) (Suppl. Table S9).

The control sessions were performed with the same timing of events as the inactivation sessions. Every experimental session started with a pre-injection testing period, followed by the injection (or a sham injection), a 30–40 min waiting period, and the post-injection testing period. We conducted 7 inactivation sessions interleaved with 7 control (no actual injection, or PBS-only injection) sessions for each monkey. In total, M1 performed 14,150 trials and M2 performed 10,945 trials in the post-injection testing period (Suppl. Table S9).

### Behavioral paradigm

Monkeys were sitting in a dark room in a custom-made primate chair with the head restrained 30 cm away from a 27″ LED display (60 Hz refresh rate, model HN274H, Acer Inc. USA). The gaze position of the right eye was monitored at 220 Hz using an MCU02 ViewPoint infrared eye tracker (Arrington Research Inc. USA). A MATLAB-based task controller (https://github.com/dagdpz/monkeypsych, MATLAB version R2012b, The MathWorks, Inc., USA) and the Psychophysics Toolbox^94^ were used to control stimulus presentation.

#### Color discrimination task

Two monkeys performed a color discrimination task (Fig. 1B) where the perceptual difficulty was determined by the color similarity of the target (T, red) vs. distractor (D, easy—yellow, difficult—orange). Target or distractor was either presented alone or with a second stimulus (distractor or target) in the opposite hemifield, determining the level of spatial competition. Monkeys had to saccade to the target or continue fixating when only distractor(s) were presented. Each trial started with the presentation of a red fixation spot. The monkey initiated each trial by acquiring eye fixation by entering the 5° radial window around the fixation spot within 500 ms after the onset of the fixation spot. After maintaining fixation for 500–900 ms, the fixation spot turned gray, and one or two peripheral dots simultaneously appeared (Go signal). Red dots represented targets, whereas yellow and orange dots represented distractors. In conditions with a single peripheral stimulus, either one target or one distractor was presented in the left or the right hemifield. In conditions with two peripheral stimuli, the monkey was shown two dots in opposite hemifields. In double-target trials, two equally-rewarded targets were presented, and the monkey could choose either target by making a saccade. In double-distractor trials, two distractors were shown, and had to be ignored by maintaining central fixation. In target-distractor trials, a target was presented with a distractor in the opposite hemifield. Together, there were 13 stimulus display configurations: single stimuli—T, D_easy_, D_diff_, stimulus is on the left or the right (6 conditions); double same stimuli—T-T, D_easy_-D_easy_, D_diff_-D_diff_ (3 conditions); double different stimuli—T-D_easy_, T-D_diff_, the target is on the left or the right (4 conditions).

Monkeys were required to make a saccade towards the target while ignoring the distractor. They had to choose within 500 ms (target acquisition epoch). As soon as the eye position entered the 5° radius window around one of the stimuli, the stimulus was selected, and the monkey was not allowed to reverse his decision. The chosen stimulus, either the selected peripheral dot for saccade responses or the fixation spot for maintaining eye fixation, turned bright to confirm selection. After the monkey fixated the selected stimulus for another 500 ms (target hold epoch), correct responses were followed by the reward tone, a fluid reward, and an inter-trial interval (ITI) of 2000 ms. Incorrect answers were followed by the error tone, no reward, and an ITI of 2000 ms.

All stimuli were matched in luminance (dim stimuli: 11 cd/m^2^, bright stimuli: 35 cd/m^2^) and size (1° diameter). Targets and distractors were displayed at one of three locations per hemifield (six locations in total) with an eccentricity of 20° of visual angle (Fig. 1B**,** inset). Stimulus locations were arranged radially around the central fixation spot at the horizontal meridian and at 20° angle above and below the horizontal meridian. In conditions involving two peripheral stimuli, the stimuli were presented symmetrically at the same vertical location (i.e., upper left—upper right; middle left—middle right; lower left—lower right) or across the diagonal axis (i.e., upper left—lower right; lower left—upper right), in equal proportions. All experimental conditions (stimulus type, difficulty level, spatial location) were pseudorandomized. Trials that had been aborted before the monkey selected a stimulus were returned to the pool of trials from which the next trial was chosen randomly.

#### Distractor color determination

After initial training of the task with an easy yellow distractor, we determined the difficult distractor color (orange) for the main experiment based on the results of a psychophysical assessment, in six pre-test sessions in each monkey. The goal of this assessment was to determine a distractor color that could be correctly discriminated from the target with 70—80% accuracy. To this end, the monkeys performed a color discrimination test with five distractor colors of different perceptual difficulty ranging from yellow (easy, RGB [60 60 0]) to red–orange (difficult, M1: [128 11 0]; M2: [128 23 0]). All trial conditions were presented in a pseudorandomized order. The perceptual difficulty was defined as the RGB value ratio between green (G) and red (R). A stimulus with a G/R ratio of 1 is a yellow distractor (60 60 0) which is perceptually very different from the target color ([128 0 0]) with a G/R ratio of 0. For the main experiment, we chose an orange color as the “difficult distractor” color (M1: G/R ratio = 0.09, M2: G/R ratio = 0.18, see Supplementary Information, Suppl. Figure S6). The corresponding accuracy values were fitted by the cumulative normal function using Palamedes toolbox^95^ in MATLAB 2019b (The MathWorks, Inc. USA). Note that we did not test all five levels of perceptual difficulty in the main experiment, but only used two levels of difficulty, to limit the number of stimulus conditions and allow enough trial repetitions in each condition.

#### Equalizing spatial choice behavior

In the beginning of each task training session, the left–right choice behavior in target-target trials was approximately equalized by shifting the entire stimulus array (both central and peripheral stimuli) with respect to the body midline, if a substantial hemifield bias was present^31^. In all subsequent experimental sessions, the stimulus array was adjusted by the same amount (0° from the midline for M1 and by 5° for M2).

### Data analysis

We analyzed the behavioral data from two monkeys for the “post-injection” testing period, with seven injections and seven control sessions for each monkey. Data analysis was performed using MATLAB. The analysis focuses on the saccade latencies, accuracy, and Signal Detection Theory variables to evaluate if there is a significant statistical difference between control and inactivation sessions. Accuracy was defined as the proportion of correct and rewarded trials among all trials in a specific condition (e.g. correct hits and rejections for all single stimuli trials, regardless of the hemifield). For calculating the saccade latencies, we used all completed saccades to the target.

### Saccade definition

All eye movements with a minimum velocity of 200°/s and a minimum duration of 30 ms were considered saccades. To detect a saccade, the instantaneous saccade velocity was calculated sample by sample as the square root of the sum of squared interpolated (220 Hz to 1 kHz) and smoothed (12 ms moving average rectangular window) horizontal and vertical eye position traces, and smoothed again (12 ms moving average rectangular window). Saccade onset was defined as the first eye position change after the go-cue that exceeded a velocity threshold of 200°/s. Saccade end was defined as the first point in time when eye velocity dropped below 50°/s after saccade onset.

### Signal detection theory

To address our two alternative hypotheses, whether the contralesional visuospatial deficit is a consequence of a contralesional perceptual discrimination deficit or spatial selection bias, we used the Signal Detection Theory to assess changes in perceptual sensitivity index (d-prime) and response criterion after unilateral reversible dorsal pulvinar inactivation. The d-prime (*d'*) measures how well the monkeys discriminate targets from distractors (Eq. 1); z represents z-score calculated using normal inverse cumulative distribution function (*norminv* function in MATLAB). The response criterion (*c*) indicates the tendency to select a stimulus in a specific hemifield regardless if it is a target or distractor (Eq. 2).

1 $${{\text{d}}}^{{{\prime}}}={\text{z}}\left(\mathrm{False \,alarm \,rate}\right)-{\text{z}}(\mathrm{Hit \,rate})$$

2 $${\text{c}} = { } - 0.5\left( {{\text{z}}\left( {\text{Hit rate}} \right) + {\text{z}}\left( {\text{False alarm rate}} \right)} \right)$$

The data were analyzed separately for each monkey (M1 and M2), for each difficulty level (yellow and orange distractor), and stimulus type (single, double same, and double different stimuli). To compare the effect of inactivation per hemifield, we calculated the signal detection theory variables separately for the contralesional and ipsilesional hemifield relative to the side of inactivation, collapsing across all stimulus positions within each hemifield (see details in the Supplementary Information, Suppl. Figures S2-S4). An increase of the criterion signifies decreased selection of the contralateral stimulus (“less contra”) and vice versa. Accuracy across both hemifields was defined as the number of hits and correct rejections divided by the total number of trials for this stimulus condition. As a control, we also performed the analysis of hits, misses, correct rejections and false alarms per target position and contrasted the resulting accuracy for the upper and the lower targets within each hemifield, but did not observe any systematic differences or large variations (Suppl. Figure S7, Suppl. Table S10).

### Statistical analysis

The statistical analysis was performed using R (version 4.1.2, R Core Team, 2022) and MATLAB 2014b. First, to assess whether the accuracy differs between the three stimulus types (single stimuli, double same, and double different stimuli) for each difficulty level in the control sessions due intended consequence of our task design, we conducted a mixed ANOVA followed by post-hoc tests to determine whether the three stimulus types differed significantly (corrected for multiple comparisons using Bonferroni correction).

The main aim of the study was to investigate the effects of dorsal pulvinar inactivation compared to control (no perturbation) sessions on different dependent variables. Accuracy was analyzed with three-way mixed ANOVAs: Difficulty level (easy, difficult) × Stimulus type (single, double same, double different) × Perturbation (inactivation, control). The d-prime and criterion were analyzed with four-way mixed ANOVAs: Difficulty level (easy, difficult) × Stimulus type (single, double same, double different) × Perturbation (inactivation, control) × Hemifield (contra, ipsi). Although the four-factor mixed ANOVA includes all possible interactions, it cannot directly answer our research question: whether dPul inactivation affects the criterion or d-prime, differently for the three stimulus types and the two perceptual difficulty levels. To assess whether there was a statistical difference between the inactivation sessions and control sessions in the d-prime and criterion, we conducted independent sample t-tests separately for the stimulus position (contralesional and ipsilesional hemifield), the stimulus type (single, double same, and double different), and the difficulty level (difficult and easy). Due to a small sample size, we also calculated non-parametric tests (Wilcoxon rank sum test), which led to comparable results (Suppl. Table S8).

### Simulations

We numerically simulated different scenarios of stimulus selection corresponding to the two alternative hypotheses (response bias and perceptual sensitivity deficit). These simulations aimed to visualize the effects of unilateral inactivation on selection behavior and resulting STD variables for each scenario, and to compare the changes derived from the predictions of each hypothesis with the data. One group of scenarios represents the response criterion hypothesis, where we expect a decrease both in contralesional hit rate and false alarm rate after the inactivation, resulting in a shift of criterion away from the contralesional hemifield—i.e. towards “less contra”. The other group of scenarios represents the perceptual discrimination hypothesis, where we expect a decrease in the contralesional hit rate and an increase in false alarm rate, resulting in decreased contralesional d-prime. In brief, the proportions of hits, misses, correct rejections, and false alarms were defined for the control condition (no inactivation), approximately based on the actual monkey performance. A specific bias or perceptual deficit on these proportions was introduced to estimate resulting hits, misses, correct rejections, and false alarms in the “inactivated” condition. The resulting criterion and d-prime values were calculated for the control and the inactivated conditions.

For each scenario, the simulated data consisted of 200 trials (100 target and 100 distractor trials for single stimuli in each hemifield, and for double same stimuli; 100 left target—right distractor trials and 100 left distractor—right target trials for double different stimuli). Trials were separated into four different outcomes: hits, misses, false alarms and correction rejections. The proportion of selection for one specific stimulus type and one difficulty level is set in each scenario. An example of a specific selection pattern is illustrated for the scenario “contralesional single stimulus—difficult discrimination—response criterion hypothesis” (Table 3). Before inactivation, the selection pattern is: hits: 0.7 fraction of target selection (correct, 70 trials), misses: 0.3 fraction staying on the central fixation spot when a target is presented (incorrect, 30 trials); correct rejections 0.6 fraction staying when a difficult distractor is presented (correct, 60 trials) and false alarms: 0.4 fraction selecting the distractor (incorrect, 40 trials). The hit rate is 0.7, and the false alarm rate is 0.4, resulting in a criterion of − 0.14 and a d-prime of 0.77. According to the prediction of response bias hypothesis, after the inactivation the monkey should select less often stimuli presented in the contralesional hemifield regardless whether a target or a distractor is shown. We therefore expect a decrease of hits and false alarms, and an increase of misses and correct rejections, resulting in a decrease of the contralesional hit rate and false alarm rate. In this example, we chose an inactivation-induced decrease in contralesional selection by 0.2 (20 trials) for the hit and false alarm rates, resulting in a shift of the criterion towards “less contra” (increase of the criterion). Notably, the d-prime is also changing slightly. To visualize how the hit rate, the false alarm rate, the criterion and the d-prime are related, we plotted the resulting values of criterion and d-prime for each combination of the false alarm rate and the hit rate, as a color-coded image.

**Table 3:** An example selection pattern for the simulated hypothetical scenario “contralesional single stimulus—difficult discrimination—response bias hypothesis”.

	Before inactivation	Inactivation effect	After inactivation
Hits	0.70	− 0.2	0.50
Misses	0.30	+ 0.2	0.50
Correct rejections	0.60	+ 0.2	0.80
False alarms	0.40	− 0.2	0.20
Hit rate	0.70	− 0.2	0.50
False alarm rate	0.40	− 0.2	0.20
Criterion	− 0.14		0.42
d-prime	0.77		0.84

This example is illustrated in Fig. 4A.

In addition to the simulations of hemifield-specific effects, we also performed simulations of “stay”, “neutral” and “go” non-hemifield-specific bias. These simulations modelled the tendency to select a peripheral stimulus, or maintain central fixation, and are linked to the non-hemifield-specific analysis of accuracy in each stimulus condition (cf. Fig. 3; Suppl. Table S7). Based on these simulations, we defined, for the double different stimuli, bias < 0.67 as “go” and bias > 0.67 as “stay”, because criterion = 0.67 corresponds to when the proportions of staying (misses) and of selecting peripheral stimulus (hits + false alarms) are equal. For the two other stimuli conditions the “go” bias corresponds to the criterion < 0.

All simulations were done in MATLAB 2014b. The code is publically available at https://github.com/dagdpz/perceptual-dis/simulations.

### Supplementary Information

Supplementary Information.

## Data Availability

The datasets generated and analyzed for the current study, and the corresponding code, are available from the corresponding author on reasonable request, and are also uploaded to a public repository at https://osf.io/aq9vw/.
