# Supplementary material for: Dorsal pulvinar inactivation leads to spatial selection bias without perceptual deficit
Source: Sci Rep. 2024 Jun 4;14:12852. doi: 10.1038/s41598-024-62056-5 (PMC11150452; doi:10.1038/s41598-024-62056-5)
Supplement: Supplementary file 1 — Supplementary Information. [file 41598_2024_62056_MOESM1_ESM.pdf]

Supplementary Information

Dorsal pulvinar inactivation leads to spatial selection bias without perceptual deficit

Kristin Kaduk, Melanie Wilke, Igor Kagan

7 Supplementary Figures

10 Supplementary Tables

Supplementary Figures

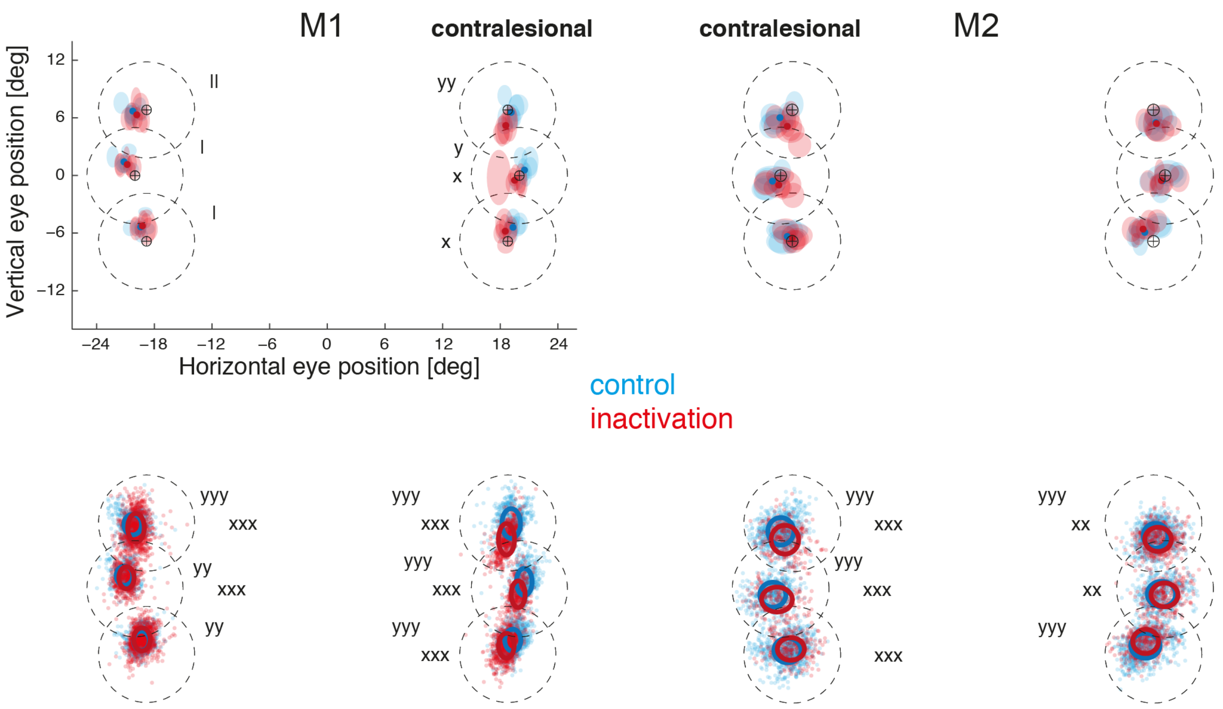

Supplementary Figure S1. Saccade endpoint accuracy and precision before and after the inactivation, for all completed saccade trials. Saccade endpoints were defined as the eye position of the end of the saccade (velocity < 50°/sec) that entered the window. Top row: session-by-session analysis. Each ellipse represents the mean saccade endpoint (center) and the standard deviations (in horizontal x and vertical y direction) as the ellipse half-axes, per session. Blue (control) and red (inactivation) dots represent means across corresponding sessions. Dashed black circles represent allowed fixation windows. Bottom row: analysis across all trials from all control (blue) and inactivation (red) sessions. Each small semitransparent dot represents an endpoint in one trial, ellipses show mean ± standard deviation (horizontal and vertical) of endpoints across trials.

To test significant differences between control and inactivation data, for the comparison across sessions, the Wilcoxon rank sum test for independed samples was used; for comparison across trials, the independent t-test was used. For accuracy: symbols **x / xx / xxx** (p<0.05 / p<0.01/ p<0.001) represent significant difference of session mean endpoints across sessions, or difference in mean across all trials, horizontally; symbols **y / yy / yyy** (p<0.05 / p<0.01 / p<0.001) represent significant difference of session mean endpoints across sessions, or difference in mean across all trials, vertically. For precision (only across sessions): symbols **- / -- / ---** (p<0.05 / p<0.01 / p<0.001) represent significant difference of the standard deviations of saccade endpoints in horizontal direction (note that this effect was not present); symbols **I / II / III** (p<0.05 / p<0.01 / p<0.001) represent significant difference of the standard deviations of saccade endpoints on vertical direction.

In both monkeys, a mild horizontal and vertical undershoot in the contralesional hemifield was most consistent and noticeable pattern (across sessions, significant only in M1; across trials, significant in both animals).

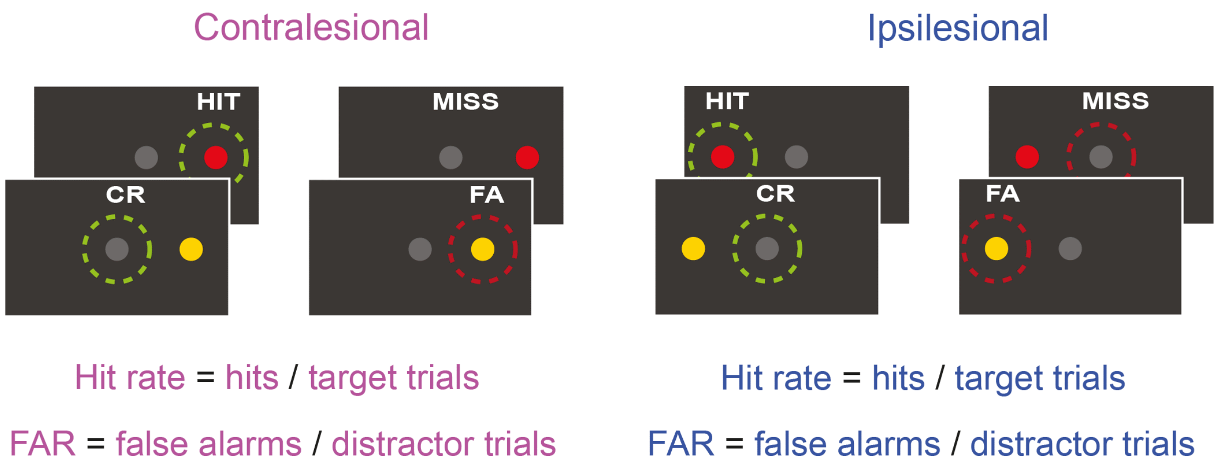

Supplementary Figure S2. Calculation of the signal detection theory variables for single stimuli related to the results in **Figure 4**. Here we describe, firstly, how trials were classified in relation to the monkey's responses and secondly, the calculations. Hits are trials where a saccade was made to a target (correct response, green dashed circle). Misses are trials where the monkey fixated the dot in the middle of the screen while a single target was displayed (incorrect response, dark red dashed circle). Correct rejections are trials where the monkey fixated the dot in the middle of the screen when a single distractor was displayed in the periphery (correct response, green dashed circle). False alarms are trials where the monkey made a saccade to the distractor (incorrect response, dark red dashed circle). We calculated the hit rate and false alarm rate (FAR) according to Hit rate = Hits / contralesional target trials and False alarm rate = False alarms / contralesional distractor trials. We used the standard calculations for the d-prime (d' = z (Hit) –z(FAR)) and criterion (c = -0.5*(z(Hit) + z(FAR))). All variables were calculated separately for stimuli presented in the ipsilesional and contralesional hemifield to compare the changes in d-prime and criterion for each hemifield.

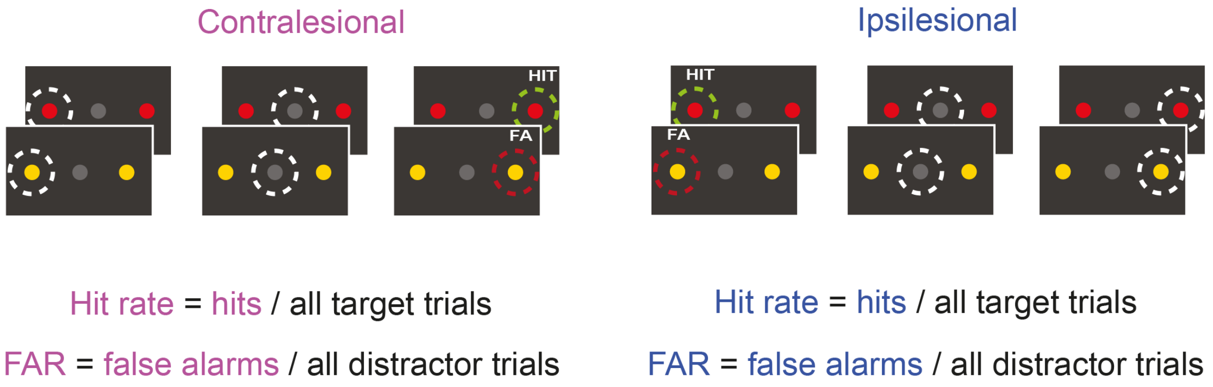

Supplementary Figure S3. Calculation of the signal detection theory variables for double same stimuli related to the results in **Figure 5**. Notations are the same as in **Suppl. Figure S2**. All variables were calculated separately for stimuli presented in the ipsilesional or contralesional hemifield, which allows us to compare the changes in d-prime and criterion for each hemifield. In the following, the examples are given for the contralesional hemifield. Contralesional hits are trials where a saccade was made to the contralesional target when a target was presented in each hemifield. Contralesional false alarms are trials where a saccade was made to the contralesional distractor when a distractor was presented in each hemifield. Hit rate is computed as all contralesional hits divided by all double same target trials. False alarm rate is computed as all contralesional false alarms divided by all double same distractor trials. We used the standard calculations for the d-prime (d' = z(Hit) –z(FAR)) and criterion (c = -0.5*(z(Hit) + z(FAR))).

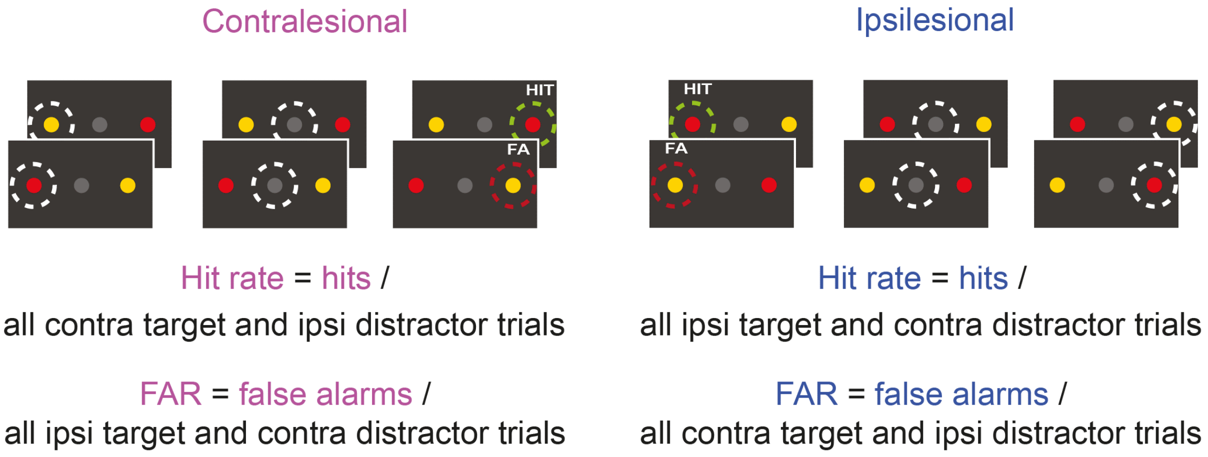

Supplementary Figure S4. Calculation of the signal detection theory variables for double different stimuli related to the results in **Figure 6**. Notations are the same as in **Suppl. Figure S2**. All variables were calculated separately for stimuli presented in the ipsilesional or contralesional hemifield, which allows us to compare the changes in d-prime and criterion for each hemifield. In the following, examples are given for the ipsilesional hemifield. Ipsilesional hits are trials where a saccade was made to the ipsilesional target. Ipsilesional false alarms are trials where a saccade was made to the ipsilesional distractor. The hit rate is computed as all ipsilesional hits divided by all double different trials where a target was presented in the ipsilesional hemifield (including all response options, i.e. fixation and saccades to either ipsi- or contralesional stimulus). Likewise, false alarm rate (FAR) is computed as all ipsilesional false alarms divided by all double different trials where a distractor was presented in the ipsilesional hemifield. We used the standard calculations for the d-prime ($d' = z(Hit) - z(FAR)$) and criterion ($c = -0.5*(z(Hit) + z(FAR))$).

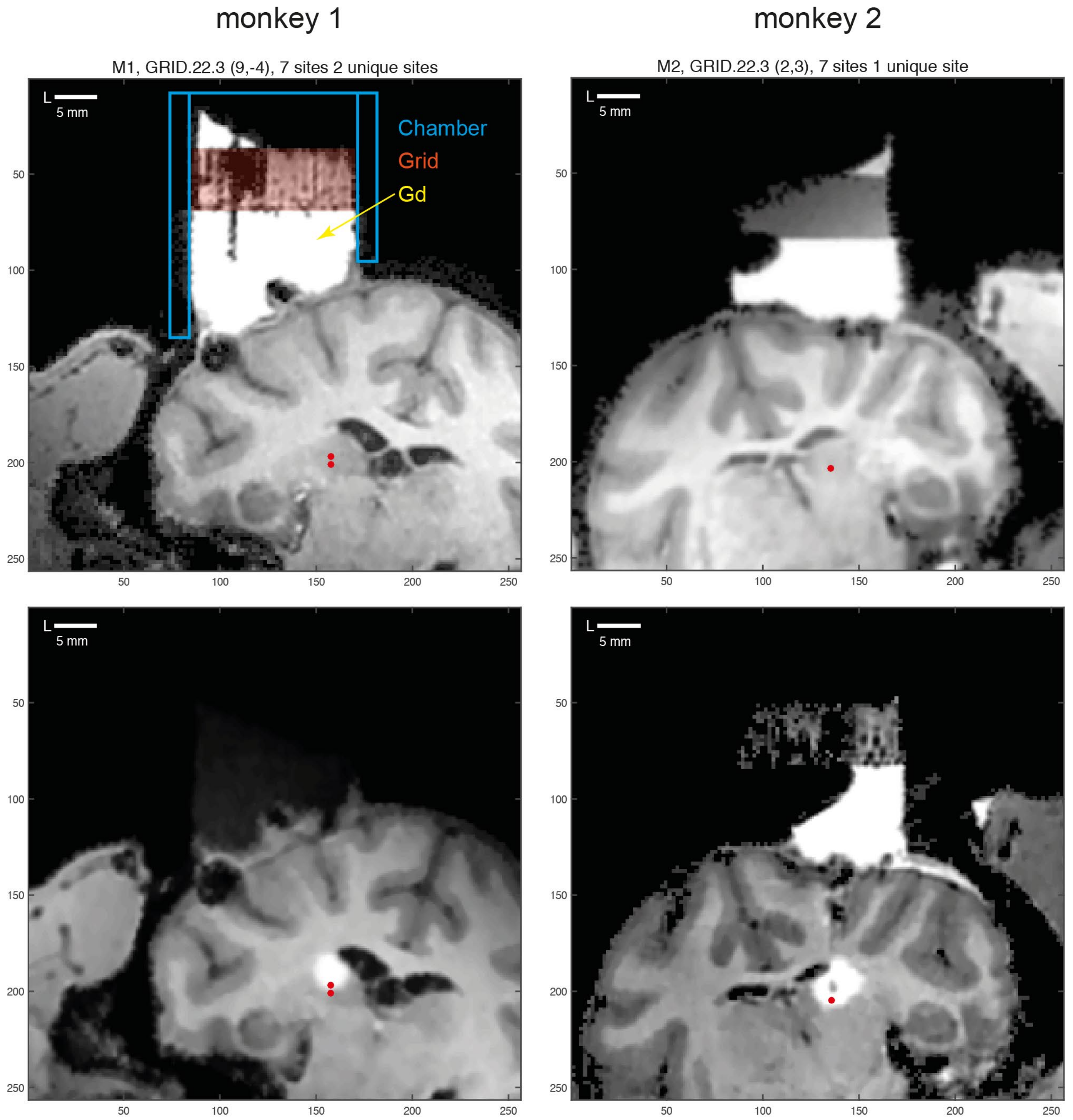

Supplementary Figure S5. Inactivation site localization, shown in T1-weighted scan sections transformed into "chamber normal" (aligned to the chamber and grid vertical axis) plane. Red dots denote injection cannula tip locations. Bottom row: T1-weighted scans with MRI contrast agent gadolinium injection, 1-2 mm shallower than the final injection sites.

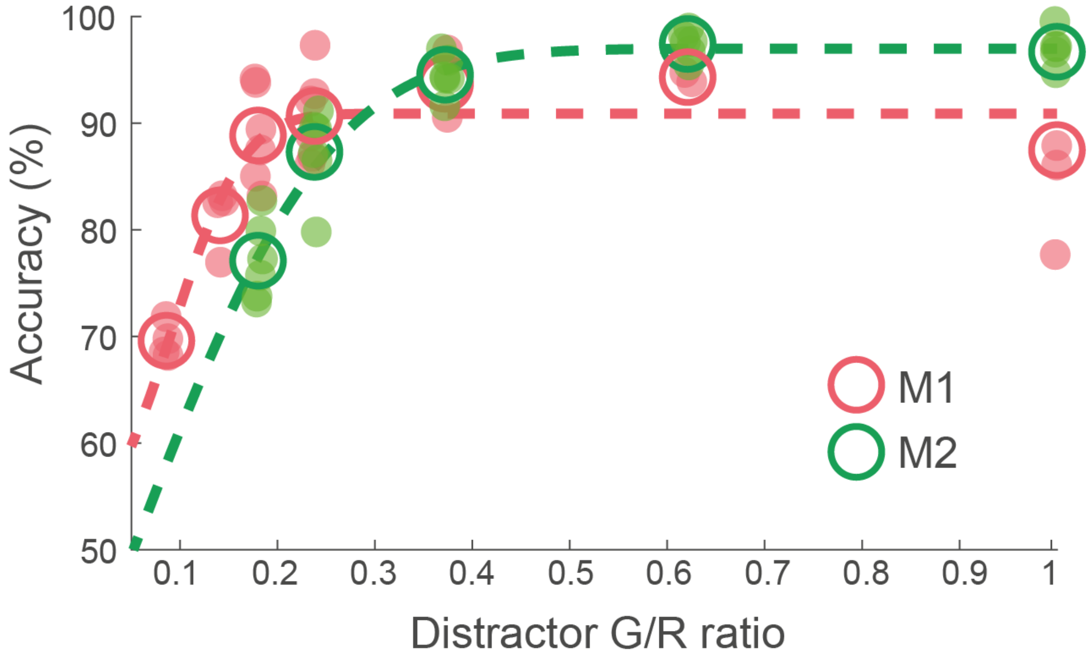

Supplementary Figure S6. Accuracy per distractor color. The monkeys performed a color discrimination paradigm with five distractor colors of different perceptual difficulty ranging from yellow (easy, G/R ratio: 1, RGB [60 60 0]) to red-orange (difficult, G/R ratio M1: 0.09, M2: 0.18, [M1: 128 11 0; M2: 128 23 0]). We calculated how accurate the target was discriminated from a distractor in the opposite hemifield. The large dots display the mean accuracy across sessions for the different applied G/R ratios separated for each monkey. To these accuracy values, the cumulative normal function was fitted. The small transparent dots display the accuracy per session. The goal of the assessment was to determine a distractor color that could be correctly discriminated from the target with 70 - 80% accuracy, for the difficult perceptual condition.

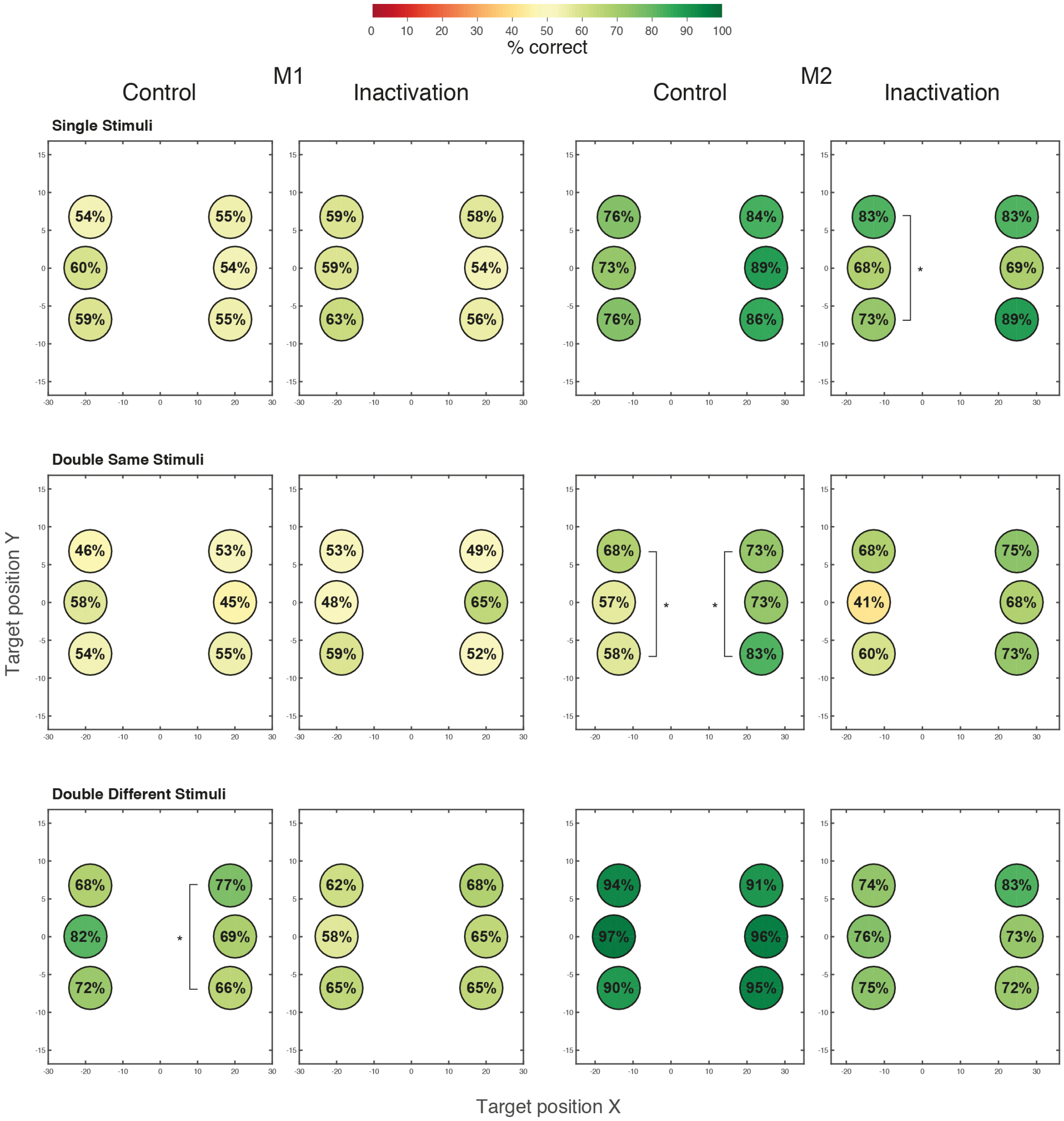

Supplementary Figure S7. Accuracy per stimulus position, separately for each stimulus condition, in control and inactivation sessions, for difficult discrimination. For single stimuli, the accuracy was computed as the number of hits and correct rejections divided by the number of all trials where a stimulus was shown at this position (hits, misses, correct rejections, false alarms). For double same stimuli, the accuracy was computed as the number of hits at this position plus correction rejections (neither of the two distractors was selected), divided by the sum of hits and false alarms at this position, plus correct rejections and misses (neither of the two targets was selected). For double different stimuli, the accuracy was computed as the number of hits at this position divided by the sum of hits and false alarms at this position, plus misses (neither of the target nor the distractor in the opposite hemifield was selected). The significance between upper and lower positions in each hemifield was assessed by the independent t-test; stars indicate $p<0.05$. See **Supplementary Table S10** for detailed statistics and for data on easy discrimination.

Supplementary Tables

Supplementary Table S1. Three-way mixed ANOVA on saccade latency, with within-factors "Stimulus Type" (single / double same / double different) and "Hemifield" (contralesional/ipsilesional) and between-factor "Perturbation" (control/inactivation sessions); ges - generalized eta squared. Significant effects are shown in bold font.

Factor	DFn	DFd	F	p-value	p<.05	ges	Monkey
**Perturbation**	**1**	**11**	**5.96**	**0.033**	*****	**0.24**	M1
**Hemifield**	**1**	**11**	**20.08**	**0.001**	*****	**0.18**	
**Stimulus Type**	**2**	**22**	**25.29**	**< .001**	*****	**0.20**	
**Perturbation × Hemifield**	**1**	**11**	**21.81**	**0.001**	*****	**0.20**	
**Perturbation × Stimulus Type**	**2**	**22**	**5.25**	**0.014**	*****	**0.05**	
Hemifield × Stimulus Type	2	22	1.12	0.344		0.02	
**Perturbation × Hemifield × Stimulus Type**	**2**	**22**	**4.64**	**0.021**	*****	**0.07**	
**Perturbation**	**1**	**12**	**7.48**	**0.018**	*****	**0.26**	M2
**Hemifield**	**1**	**12**	**6.72**	**0.024**	*****	**0.15**	
**Stimulus Type**	**2**	**24**	**14.19**	**< .001**	*****	**0.08**	
Perturbation × Hemifield	1	12	3.47	0.087		0.08	
Perturbation × Stimulus Type	2	24	1.27	0.299		0.01	
**Hemifield × Stimulus Type**	**2**	**24**	**11.44**	**< .001**	*****	**0.05**	
**Perturbation × Hemifield × Stimulus Type**	**2**	**24**	**3.74**	**0.039**	*****	**0.02**	

Supplementary Table S2. Pairwise t-tests comparing the accuracy in the different stimulus type conditions for the control sessions, separately within each perceptual difficulty (difficult/easy discrimination). Significant effects are shown in bold font.

Comparison	Difficulty	95% Confidence Intervals	t-value	p-value	p<.05	Monkey
Single Stimuli vs. Double Same Stimuli	difficult	**7.85, 21.97**	**14.91**	**< .001**	*	M1
Single Stimuli vs. Double Different Stimuli	difficult	**-20.54, -6.41**	**-13.47**	**< .001**	*	
Double Same Stimuli vs. Double Different	difficult	**-35.45, -21.30**	**-28.39**	**< .001**	*	
Single Stimuli vs. Double Same Stimuli	easy	-3.66, 0.93	-1.36	.4		
Single Stimuli vs. Double Different Stimuli	easy	-2.63, 1.94	-0.34	.9		
Double Same Stimuli vs. Double Different	easy	-1.28, 3.31	1.02	.7		
Single Stimuli vs. Double Same Stimuli	difficult	**1.92, 8.17**	**5.05**	**.001**	*	M2
Single Stimuli vs. Double Different Stimuli	difficult	**-16.91, -10.65**	**-13.79**	**< .001**	*	
Double Same Stimuli vs. Double Different	difficult	**-21.96, -15.71**	**-18.83**	**< .001**	*	
Single Stimuli vs. Double Same Stimuli	easy	-5.96, 2.35	-1.81	.8		
Single Stimuli vs. Double Different Stimuli	easy	-5.36, 2.96	-1.20	.9		
Double Same Stimuli vs. Double Different	easy	-3.56, 4.77	0.60	.9		

Supplementary Table S3. Three-way mixed ANOVA on accuracy, with within-factors “Stimulus Type” (single / double same / double different and “Difficulty” (difficult/easy discrimination) and between-factor “Perturbation” (control/inactivation sessions); ges - generalized eta squared. Significant effects are shown in bold font.

Factor	DFn	DFd	F	p-value	p<.05	ges	Monkey
Perturbation	1	12	1.46	0.250		0.06	
**Difficulty**	**1**	**12**	**349.71**	**< .001**	*****	**0.87**	
**Stimulus Type**	**2**	**24**	**6.09**	**.007**	*****	**0.11**	
Perturbation × Difficulty	1	12	1.36	.275		0.03	M1
Perturbation × Stimulus Type	2	24	3.17	.060		0.06	
**Difficulty × Stimulus Type**	**2**	**24**	**124.01**	**< .001**	*****	**0.29**	
Perturbation × Difficulty × Stimulus Type	2	24	1.88	.175		0.01	
**Perturbation**	**1**	**12**	**9.42**	**.010**	*****	**0.22**	
**Difficulty**	**1**	**12**	**421.89**	**< .001**	*****	**0.72**	
**Stimulus Type**	**2**	**24**	**8.12**	**.002**	*****	**0.24**	
Perturbation × Difficulty	1	12	0.07	.798		0.0004	M2
**Perturbation × Stimulus Type**	**2**	**24**	**6.40**	**.006**	*****	**0.20**	
**Difficulty × Stimulus Type**	**2**	**24**	**104.36**	**< .001**	*****	**0.48**	
Perturbation × Difficulty × Stimulus Type	2	24	1.12	.342		0.01	

Supplementary Table S4. Four-way mixed ANOVA on d-prime and criterion with within-factor "Stimulus type" (single / double same / double different), "Difficulty" (easy / difficult), and "Hemifield" (contralesional / ipsilesional) and between-factor "Perturbation" (control / inactivation sessions), separately for each stimulus type, for the difficult discrimination; ges - generalized eta squared. Significant effects are shown in bold font, effects involving perturbation factor are highlighted with gray background.

Factor	DFn	DFd	F	p-value	p<.05	ges	DV	Monkey
Perturbation	1	12	3.62	.081		0.18	criterion	M1
Hemifield	1	12	1.28	.281		0.01		
Difficulty	1	12	0.56	.467		0.00		
**Stimulus Type**	**2**	**24**	**7.14**	**.004**	*	**0.02**		
Perturbation × Hemifield	1	12	2.70	.127		0.03		
**Perturbation × Difficulty**	**1**	**12**	**17.86**	**.001**	*	**0.02**		
**Perturbation × Stimulus Type**	**2**	**24**	**17.30**	**< .001**	*	**0.05**		
**Hemifield × Difficulty**	**1**	**12**	**611.37**	**< .001**	*	**0.43**		
**Hemifield × Stimulus Type**	**2**	**24**	**220.56**	**< .001**	*	**0.48**		
Difficulty × Stimulus Type	2	24	2.71	.087		0.00		
Perturbation × Hemifield × Difficulty	1	12	0.09	.767		0.00		
Perturbation × Hemifield × Stimulus Type	2	24	1.35	.279		0.01		
**Perturbation × Difficulty × Stimulus Type**	**2**	**24**	**8.69**	**.001**	*	**0.01**		
**Hemifield × Difficulty × Stimulus Type**	**2**	**24**	**47.64**	**< .001**	*	**0.14**		
Perturbation × Hemifield × Difficulty × Stimulus Type	2	24	8.69	.116		0.01		
Perturbation	1	12	1.42	.257		0.03	d-prime	
Hemifield	1	12	3.98	.069		0.03		
**Difficulty**	**1**	**12**	**227.69**	**< .001**	*	**0.81**		
**Stimulus Type**	**2**	**24**	**105.16**	**< .001**	*	**0.55**		
Perturbation × Hemifield	1	12	4.61	.053		0.03		
Perturbation × Difficulty	1	12	0.82	.383		0.02		
Perturbation × Stimulus Type	2	24	1.75	.196		0.02		
Hemifield × Difficulty	1	12	0.07	.796		0.00		
Hemifield × Stimulus Type	2	24	0.82	.452		0.01		
**Difficulty × Stimulus Type**	**2**	**24**	**19.85**	**< .001**	*	**0.10**		
Perturbation × Hemifield × Difficulty	1	12	2.26	.159		0.01		
**Perturbation × Hemifield × Stimulus Type**	**2**	**24**	**3.99**	**.032**	*	**0.03**		
Perturbation × Difficulty × Stimulus Type	2	24	0.81	.459		0.01		
Hemifield × Difficulty × Stimulus Type	2	24	3.13	.062		0.01		
**Perturbation × Hemifield × Difficulty × Stimulus Type**	**2**	**24**	**3.87**	**.035**	*	**0.01**		

**Perturbation**	**1**	**12**	**12.14**	**.005**	*	**0.28**	criterion	M2
**Hemifield**	**1**	**12**	**102.84**	**< .001**	*	**0.57**		
Difficulty	1	12	0.56	.469		< 0.01		
**Stimulus Type**	**2**	**24**	**7.99**	**.002**	*	**0.08**		
**Perturbation × Hemifield**	**1**	**12**	**6.71**	**.024**	*	**0.08**		
Perturbation × Difficulty	1	12	0.39	.547		< 0.01		
**Perturbation × Stimulus Type**	**2**	**24**	**7.82**	**.002**	*	**0.08**		
**Hemifield × Difficulty**	**1**	**12**	**542.18**	**< .001**	*	**0.60**		
**Hemifield × Stimulus Type**	**2**	**24**	**202.10**	**< .001**	*	**0.64**		
**Difficulty × Stimulus Type**	**2**	**24**	0.82	.454		0.01		
Perturbation × Hemifield × Difficulty	1	12	1.03	.330		< 0.01		
Perturbation × Hemifield × Stimulus Type	2	24	2.22	.130		0.02		
Perturbation × Difficulty × Stimulus Type	2	24	0.74	.486		0.01		
**Hemifield × Difficulty × Stimulus Type**	**2**	**24**	**104.88**	**< .001**	*	**0.22**		
Perturbation × Hemifield × Difficulty × Stimulus Type	2	24	0.33	.723		< 0.01		
**Perturbation**	**1**	**12**	**10.00**	**.008**	*	**0.29**	d-prime	
**Hemifield**	**1**	**12**	**41.40**	**< .001**	*	**0.69**		
**Difficulty**	**1**	**12**	**591.40**	**< .001**	*	**0.77**		
**Stimulus Type**	**2**	**24**	**179.90**	**< .001**	*	**0.02**		
Perturbation × Hemifield	1	12	2.24	.160		0.01		
Perturbation × Difficulty	1	12	0.82	.382		0.14		
**Perturbation × Stimulus Type**	**2**	**24**	**8.76**	**.001**	*	**0.01**		
Hemifield × Difficulty	1	12	0.19	.674		0.14		
**Hemifield × Stimulus Type**	**2**	**24**	**18.44**	**< .001**	*	**0.08**		
**Difficulty × Stimulus Type**	**2**	**24**	**21.91**	**< .001**	*	**0.01**		
Perturbation × Hemifield × Difficulty	1	12	2.33	.153		0.01		
Perturbation × Hemifield × Stimulus Type	2	24	1.11	.346		0.01		
Perturbation × Difficulty × Stimulus Type	2	24	0.26	.774		< 0.01		
**Hemifield × Difficulty × Stimulus Type**	**2**	**24**	**3.48**	**.047**	*	**0.02**		
Perturbation × Hemifield × Difficulty × Stimulus Type	2	24	0.33	.721		< 0.01		

Supplementary Table S5. Two-way mixed ANOVA on d-prime and criterion with within-factor "Hemifield" (contralesional/ipsilesional) and between-factor "Perturbation" (control/inactivation sessions), separately for each stimulus type, **for difficult discrimination**; ges - generalized eta squared. Significant effects are shown in bold font.

Factor	DFn	DFd	F	p-value	p<.05	ges	DV	Monkey	Stimulus type
Perturbation	1	12	0.70	.42		0.03	criterion	M1	Single
**Hemifield**	**1**	**12**	**98.70**	**< .001**	*	**0.80**			
Perturbation × Hemifield	1	12	2.23	.161		0.08			
Perturbation	1	12	0.31	.588		0.01	d-prime		
Hemifield	1	12	3.77	.076		0.15			
Perturbation × Hemifield	1	12	0.003	.96		< .001			
**Perturbation**	**1**	**12**	**6.40**	**.026**	*	**0.17**	criterion	M2	
**Hemifield**	**1**	**12**	**69.56**	**< .001**	*	**0.78**			
Perturbation × Hemifield	1	12	3.09	.104		0.14			
Perturbation	1	12	3.46	.087		0.18	d-prime		
**Hemifield**	**1**	**12**	**33.58**	**< .001**	*	**0.39**			
Perturbation × Hemifield	1	12	0.68	.425		0.013			
**Perturbation**	**1**	**12**	**9.65**	**.009**	*	**0.42**	criterion	M1	Double Same
Hemifield	1	12	2.19	.165		0.02			
Perturbation × Hemifield	1	12	1.11	.313		0.01			
Perturbation	1	12	0.76	.401		0.01	d-prime		
Hemifield	1	12	1.42	.256		0.09			
Perturbation × Hemifield	1	12	2.89	.115		0.17			
**Perturbation**	**1**	**12**	**10.43**	**.007**	*	**0.44**	criterion	M2	
**Hemifield**	**1**	**12**	**82.07**	**< .001**	*	**0.43**			
Perturbation × Hemifield	1	12	2.62	.132		0.02			
Perturbation	1	12	0.02	.888		0.00	d-prime		
**Hemifield**	**1**	**12**	**55.80**	**< .001**	*	**0.77**			
Perturbation × Hemifield	1	12	1.95	.188		0.11			
**Perturbation**	**1**	**12**	**6.47**	**.026**	*	**0.34**	criterion	M1	Double Different
Hemifield	1	12	4.04	.067		0.01			
Perturbation × Hemifield	1	12	1.86	.198		0.00			
Perturbation	1	12	2.91	.114		0.19	d-prime		
Hemifield	1	12	0.90	.361		0.00			
Perturbation × Hemifield	1	12	0.32	.582		0.00			
Perturbation	1	12	1.53	.24		0.10	criterion	M2	
**Hemifield**	**1**	**12**	**26.77**	**< .001**	*	**0.27**			
**Perturbation × Hemifield**	**1**	**12**	**6.88**	**.022**	*	**0.09**			
**Perturbation**	**1**	**12**	**10.73**	**.007**	*	**0.46**	d-prime		
Hemifield	1	12	1.23	.289		< 0.01			
Perturbation × Hemifield	1	12	< 0.01	.993		< 0.01			

Supplementary Table S6. Two-way mixed ANOVA on d-prime and criterion with within-factor "Hemifield" (contralesional/ipsilesional) and between-factor "Perturbation" (control/inactivation sessions), separately for each stimulus type, **for easy discrimination**; ges - generalized eta squared. Significant effects are shown in bold font.

Factor	DFn	DFd	F	p-value	p<.05	ges	DV	Monkey	Stimulus type
Perturbation	1	12	0.37	.557		0.02	criterion	M1	Single
Hemifield	1	12	0.74	.405		0.02	criterion	M1	Single
Perturbation × Hemifield	1	12	1.14	.306		0.03	criterion	M1	Single
Perturbation	1	12	2.09	.174		0.12	d-prime	M1	Single
Hemifield	1	12	0.84	.378		0.01	d-prime	M1	Single
Perturbation × Hemifield	1	12	1.16	.302		0.02	d-prime	M1	Single
Perturbation	1	12	1.73	.213		0.06	criterion	M2	Single
**Hemifield**	**1**	**12**	**28.33**	**< .001**	*	**0.58**	criterion	M2	Single
Perturbation × Hemifield	1	12	4.59	.053		0.18	criterion	M2	Single
Perturbation	1	12	3.50	.086		0.16	d-prime	M2	Single
Hemifield	1	12	4.34	.059		0.11	d-prime	M2	Single
Perturbation × Hemifield	1	12	1.28	.279		0.04	d-prime	M2	Single
**Perturbation**	**1**	**12**	**8.30**	**.014**	*	**0.39**	criterion	M1	Double Same
**Hemifield**	**1**	**12**	**832.64**	**< .001**	*	**0.87**	criterion	M1	Double Same
Perturbation × Hemifield	1	12	0.62	.445		0.01	criterion	M1	Double Same
Perturbation	1	12	0.02	.899		0.001	d-prime	M1	Double Same
Hemifield	1	12	2.56	.135		0.11	d-prime	M1	Double Same
**Perturbation × Hemifield**	**1**	**12**	**11.99**	**.005**	*	**0.37**	d-prime	M1	Double Same
**Perturbation**	**1**	**12**	**31.20**	**< .001**	*	**0.64**	criterion	M2	Double Same
**Hemifield**	**1**	**12**	**1252.30**	**< .001**	*	**0.97**	criterion	M2	Double Same
Perturbation × Hemifield	1	12	0.004	.949		< 0.01	criterion	M2	Double Same
Perturbation	1	12	2.05	.178		0.04	d-prime	M2	Double Same
**Hemifield**	**1**	**12**	**18.84**	**< .001**	*	**0.54**	d-prime	M2	Double Same
Perturbation × Hemifield	1	12	1.72	.215		0.09	d-prime	M2	Double Same
Perturbation	1	12	0.49	.498		0.03	criterion	M1	Double Different
**Hemifield**	**1**	**12**	**32.22**	**< .001**	*	**0.38**	criterion	M1	Double Different
Perturbation × Hemifield	1	12	3.93	071		0.07	criterion	M1	Double Different
Perturbation	1	12	0.98	.342		0.07	d-prime	M1	Double Different
Hemifield	1	12	0.46	.511		< 0.01	d-prime	M1	Double Different
Perturbation × Hemifield	1	12	0.05	.823		< 0.01	d-prime	M1	Double Different
**Perturbation**	**1**	**12**	**22.96**	**< .001**	*	**0.51**	criterion	M2	Double Different
**Hemifield**	**1**	**12**	**318.79**	**< .001**	*	**0.92**	criterion	M2	Double Different
**Perturbation × Hemifield**	**1**	**12**	**9.52**	**.009**	*	**0.27**	criterion	M2	Double Different
**Perturbation**	**1**	**12**	**13.49**	**.003**	*	**0.47**	d-prime	M2	Double Different
Hemifield	1	12	4.24	.062		0.07	d-prime	M2	Double Different
Perturbation × Hemifield	1	12	1.94	.189		0.03	d-prime	M2	Double Different

Supplementary Table S7. Non-hemifield-specific bias ("stay" – central fixation option vs. "go" – saccade to a peripheral stimulus) in control and inactivation sessions. For single stimuli and double same stimuli, criterion < 0 indicated a "go" bias. For double different stimuli, criterion < 0.67 indicated a "go" bias. The table shows the results of the two-sided t-test against the corresponding neutral criterion (0 or 0.67) for criterion values across sessions.

Monkey	Stimulus type	Difficulty	Session type	Criterion	t-value	p-value	Bias
M1	single stimuli	difficult	control	-1.21	-11.16	< .001	Go
			inactivation	-0.89	-4.73	.003	Go
		easy	control	0.01	0.13	.903	Neutral
			inactivation	0.10	1.46	.196	Neutral
	double same stimuli	difficult	control	-2.25	-27.63	< .001	Go
			inactivation	-2.17	-14.83	< .001	Go
		easy	control	-0.54	-10.33	< .001	Go
			inactivation	-0.49	-7.05	< .001	Go
	double different stimuli	difficult	control	0.01	-214.54	< .001	Go
			inactivation	0.02	-79.27	< .001	Go
		easy	control	0.23	-16.08	< .001	Go
			inactivation	0.41	-3.47	.013	Go
M2	single stimuli	difficult	control	-0.65	-5.8	.001	Go
			inactivation	-0.38	-6.5	.001	Go
		easy	control	0.15	2.25	.065	Neutral
			inactivation	0.50	6.39	.001	Stay
	double same stimuli	difficult	control	-1.36	-19.69	< .001	Go
			inactivation	-1.04	-9.96	< .001	Go
		easy	control	-0.18	-2.14	.076	Neutral
			inactivation	0.09	1.04	.340	Neutral
	double different stimuli	difficult	control	0.08	-20.72	< .001	Go
			inactivation	0.27	-5.54	.001	Go
		easy	control	0.41	-7.11	< .001	Go
			inactivation	0.61	-0.96	.375	Neutral

Supplementary Table S8. Nonparametric tests (Wilcoxon rank sum test) on d-prime and criterion separately for each stimulus type, hemifield and difficulty level. Same as **Table 2**, but nonparametric. Significant effects are in bold font, consistent effects across the two monkeys are highlighted with gray background.

Stimulus type	Difficulty	Measure	Hemifield	Monkey 1			Monkey 2		
				Wilcoxon rank sum	p-value	Direction of the effect	Wilcoxon rank sum	p-value	Direction of the effect
Single stimuli	difficult	criterion	contra	44	.318	-	**34**	**.017**	**Less contra**
			ipsi	45	.383	-	53	.999	-
		d-prime	contra	59	.456	-	61	.318	-
			ipsi	57	.620	-	67	.073	-
	easy	criterion	contra	47	.535	-	**36**	**.038**	**Less contra**
			ipsi	48	.620	-	44	.318	-
		d-prime	contra	64	.165	-	**70**	**.026**	**Decrease**
			ipsi	61	.318	-	60	.383	-
Double same stimuli	difficult	**criterion**	**contra**	**73**	**.007**	**Less contra**	**74**	**.004**	**Less contra**
			**ipsi**	**32**	**.007**	**Less contra**	**28**	**.001**	**Less contra**
		d-prime	contra	61	.318	-	63	.209	-
			ipsi	39	.097	-	44	.318	-
	easy	**criterion**	**contra**	**72**	**.011**	**Less contra**	**75**	**.002**	**Less contra**
			**ipsi**	**32**	**.007**	**Less contra**	**28**	**.001**	**Less contra**
		d-prime	contra	68	.053	-	**69**	**.038**	**Decrease**
			**ipsi**	**32**	**.007**	**Increase**	52	.999	-
Double different stimuli	difficult	**criterion**	**contra**	**34**	**.017**	**Less contra**	**74**	**.004**	**Less contra**
			**ipsi**	**73**	**.007**	**Less contra**	**52**	.999	-
		d-prime	contra	58	.535	-	**73**	**.007**	**Decrease**
			ipsi	62	.259	-	**73**	**.007**	**Decrease**
	easy	criterion	contra	44	.318	-	**75**	**.002**	**Less contra**
			ipsi	44	.318	-	63	.209	-
		d-prime	contra	57	.259	-	**75**	**.002**	**Decrease**
			ipsi	62	.620	-	68	.053	-

Supplementary Table S9. Information about experimental sessions.

Monkey	Session	Experiment	Hemisphere	Substance	X grid	Y grid	Depth from the top of the grid (mm)	Volume (µl)	N trials
M1	20190121	Control	-	-	-	-	-	-	1536
M1	20190131	Control	-	-	-	-	-	-	662
M1	20190213	Control	-	-	-	-	-	-	960
M1	20190216	Control	-	-	-	-	-	-	713
M1	20190227	Control	-	-	-	-	-	-	1152
M1	20190304	Control	-	-	-	-	-	-	960
M1	20190313	Control	-	-	-	-	-	-	1230
M1	20190124	Inactivation	left	THIP	9	-4	40	4.5	1816
M1	20190129	Inactivation	left	THIP	9	-4	40	5	761
M1	20190201	Inactivation	left	THIP	9	-4	40	4.6	608
M1	20190207	Inactivation	left	THIP	9	-4	40	5	988
M1	20190214	Inactivation	left	THIP	9	-4	41	5	911
M1	20190228	Inactivation	left	THIP	9	-4	41	5	986
M1	20190314	Inactivation	left	THIP	9	-4	41	5	867
M2	20190802	Control	-	-	-	-	-	-	960
M2	20190808	Control	-	-	-	-	-	-	1152
M2	20190806	Control	-	-	-	-	-	-	1237
M2	20190815	Control	-	-	-	-	-	-	961
M2	20190903	Control	right	PBS	2	3	38	0.15	792
M2	20190910	Control	right	PBS	2	3	38	0.15	1292
M2	20190912	Control	right	PBS	2	3	38	0.15	871
M2	20190729	Inactivation	right	THIP	2	3	38	0.2	394
M2	20190801	Inactivation	right	THIP	2	3	38	0.15	239
M2	20190809	Inactivation	right	THIP	2	3	38	0.15	320
M2	20190814	Inactivation	right	THIP	2	3	38	0.15	561
M2	20190820	Inactivation	right	THIP	2	3	38	0.15	618
M2	20190905	Inactivation	right	THIP	2	3	38	0.15	760
M2	20190913	Inactivation	right	THIP	2	3	38	0.15	788

Supplementary Table S10. Accuracy comparison for upper vs. lower targets in each hemifield, for control and inactivation sessions. Independent t-test was used to compare accuracy across sessions. Significant effects are in bold font. Related to **Suppl. Figure S9**.

Monkey	Stimulus type	Difficulty	Experiment	Hemifield	p-value	p<.05	t-value	Accuracy upper	Accuracy lower
M1	Single stimuli	difficult	Control	right	0.772		-0.3	0.55	0.55
				left	0.092		-2.01	0.54	0.59
			Inactivation	right	0.727		0.36	0.58	0.56
				left	0.486		-0.74	0.59	0.63
		easy	Control	right	**0.031**	*	**-2.45**	**0.89**	**0.96**
				left	0.082		2.08	0.95	0.91
			Inactivation	right	0.56		-0.6	0.83	0.88
				left	0.74		0.35	0.9	0.88
	Double same stimuli	difficult	Control	right	0.753		-0.32	0.53	0.55
				left	0.182		-1.51	0.46	0.54
			Inactivation	right	0.723		-0.36	0.49	0.52
				left	0.152		-1.64	0.53	0.59
		easy	Control	right	0.743		0.34	0.97	0.97
				left	0.092		-2	0.94	0.97
			Inactivation	right	**0.029**	*	**-2.48**	**0.95**	**0.99**
				left	0.428		0.85	0.96	0.94
	Double different stimuli	difficult	Control	right	**0.001**	*	**5.3**	**0.77**	**0.66**
				left	0.306		-1.12	0.68	0.72
			Inactivation	right	0.833		0.22	0.68	0.65
				left	0.42		-0.87	0.62	0.65
		easy	Control	right	0.06		-2.07	0.89	0.97
				left	0.971		-0.04	0.95	0.95
			Inactivation	right	0.63		-0.49	0.73	0.82
				left	0.472		0.77	0.89	0.87
M2	Single stimuli	difficult	Control	right	0.634		-0.49	0.84	0.86
				left	0.896		0.14	0.76	0.76
			Inactivation	right	0.114		-1.7	0.83	0.89
				left	**0.029**	*	**2.85**	**0.83**	**0.73**
		easy	Control	right	0.224		-1.28	0.95	0.97
				left	0.178		1.53	0.97	0.94
			Inactivation	right	0.625		-0.5	0.95	0.97
				left	0.114		1.85	0.93	0.85
	Double same stimuli	difficult	Control	right	**0.035**	*	**-2.38**	**0.73**	**0.83**
				left	*** * 0.018**	*	**3.24**	**0.68**	**0.58**
			Inactivation	right	0.708		0.38	0.75	0.73
				left	0.165		1.58	0.68	0.6
		easy	Control	right	**0.016**	*	**-2.8**	**0.97**	**1**
				left	0.387		0.93	0.98	0.97
			Inactivation	right	0.57		0.58	0.96	0.94
				left	0.435		0.84	0.97	0.93
	Double different stimuli	difficult	Control	right	0.149		-1.54	0.91	0.95
				left	0.233		1.33	0.94	0.9
			Inactivation	right	0.327		1.02	0.83	0.72
				left	0.756		-0.32	0.74	0.75
		easy	Control	right	**0.002**	*	**-4.05**	**0.94**	**0.99**
				left	0.492		0.73	0.97	0.95
			Inactivation	right	0.755		-0.32	0.9	0.92
				left	0.069		2.21	0.84	0.69
